# Spermidine enhances the heat tolerance of *Ganoderma lucidum* by promoting mitochondrial respiration driven by fatty acid β-oxidation

**DOI:** 10.1128/aem.00979-24

**Published:** 2025-01-29

**Authors:** Xiaofei Han, Zi Wang, Lingyan Shi, Ziyang Wei, Jiaolei Shangguan, Liang Shi, Mingwen Zhao

**Affiliations:** 1 Key Laboratory of Agricultural Environmental Microbiology, Ministry of Agriculture, Department of Microbiology, College of Life Sciences, Nanjing Agricultural University 70578 , Nanjing, Jiangsu, China; 2 School of Medicine, Henan Polytechnic University 12561 , Jiaozuo, Henan, China; Kyoto University, Kyoto, Japan

**Keywords:** polyamines, abiotic stress, mitochondrial respiration, fatty acid β-oxidation, heat tolerance

## Abstract

High temperature is an unavoidable environmental stress that generally exerts detrimental effects on organisms and has widespread effects on metabolism. Spermidine is an important member of the polyamines family and is involved in a range of abiotic stress responses in plants. Mitochondria play an essential role in cellular homeostasis and are key components of the stress response. Our results indicated that mitochondrial respiratory intensity increased by 80% in wild-type (WT) under heat stress, but the activities of key enzymes of the tricarboxylic acid (TCA) cycle and electron transport chain (ETC) were significantly reduced upon the knockdown of the spermidine synthase gene (*spdS*). Furthermore, the content of mitochondrial pyruvate decreased by 36.1%, whereas the levels of free fatty acid increased by 28.8% under heat stress. Upon *spdS* knockdown, the content of mitochondrial pyruvate was similar to that in the WT, but the medium-chain fatty acid (C6:0) decreased by 68.6%–84.2%, whereas the long-chain fatty acid (C18:2) marginally increased. Subsequent studies demonstrated that spermidine promoted the translation of long chain acyl-CoA dehydrogenase (LCAD) and mitochondrial trifunctional protein (MTP, also known as HADH), thereby enhancing fatty acid β-oxidation under heat stress. In conclusion, spermidine enhances key TCA cycle and ETC enzyme activities and is involved in heat stress-induced fatty acid β-oxidation by promoting the translation of LCAD and HADH, thereby improving the heat tolerance of *Ganoderma lucidum*.

**IMPORTANCE:**

Polyamines are stress-responsive molecules that enhance the tolerance of plants to multiple abiotic stresses by regulating a variety of biological processes. Our previous research indicated that heat stress induces the the biosynthesis of polyamines and promotes the conversion of putrescine to spermidine in *G. lucidum*, but the physiological role of elevated spermidine levels is yet to be elucidated. In this study, our findings demonstrated that spermidine enhances the heat tolerance in *G. lucidum* and that mitochondrial respiration is essential for spermidine-enhanced heat tolerance. This study elucidated a preliminary mechanism by which spermidine enhances heat tolerance of *G. lucidum* and provided a new insight into the understanding of how microorganisms resist heat stress.

## INTRODUCTION

The internal environment of living organisms is intricately linked to external conditions. Amidst global climate warming, high temperature has become an unavoidable environmental factor that influences various physiological processes involved in growth, metabolism, and development (1–3). Investigating the mechanism of heat response aids in elucidating the metabolic pattern of organisms under heat stress and enhancing their heat tolerance through artificial metabolic intervention. In plants, the effects of heat stress and the associated tolerance mechanisms are well-documented. For example, during the flowering stage, heat stress severely decreases the rice seed-setting rate, and rice resists heat stress through the abscisic acid (ABA) signaling pathway (4). Prolonged moderate heat stress triggers programmed cell death in tomato fruits, where cell wall invertase enhances heat tolerance by suppressing reactive oxygen species (ROS)-independent cell death (5). In microorganisms, heat stress profoundly impacts growth and metabolism, with resistance to heat stress-mediated through alterations in intracellular metabolism. For instance, heat stress induces cell death in *Saccharomyces cerevisiae* (6), with resistance to such stress conferred by Atg32-dependent mitophagy (7). In *Metarhizium robertsii*, heat stress markedly suppresses mycelial growth, whereas the fungus enhances heat stress tolerance through pyruvate accumulation (8). However, research on the heat tolerance mechanism among the majority of microbes remains limited. Investigating the heat tolerance mechanisms in fungi advances our comprehension of the metabolic responses of microorganisms to heat stress.

Polyamines are crucial modulators of stress responses that are involved in the regulation of a variety of biological processes (9). In plants, various abiotic stresses, such as heat, cold, drought, and salinity, lead to the accumulation of polyamines (10). Spermidine is an important member of the polyamines family and is instrumental in multiple stress tolerance (11). In plants, spermidine augments the heat stress resistance by regulating a series of intracellular metabolic pathways. For example, spermidine increases the heat tolerance in rice seeds by modulating endogenous starch and polyamine metabolism (12) and enhances the heat tolerance in white clover by regulating γ-aminobutyric acid (GABA) content and metabolism (13). However, the impact and underlying mechanisms of spermidine on heat stress tolerance in fungi have rarely been explored.

Mitochondria play an essential role in cellular homeostasis and are key components of the stress response (14, 15). Mitochondrial respiration generates ATP to fuel defense mechanisms and serves as a source of carbon intermediates for secondary metabolism (15). In *Arabidopsis thaliana*, the knockdown of the mitochondrial ATP synthase subunit D leads to reduced ATP production and heat tolerance (16). A wealth of research indicates that polyamines are closely associated with mitochondrial function (17, 18). Thus, a potential regulatory pathway may exist in organisms for spermidine and/or other polyamines to enhance heat tolerance via the modulation of mitochondrial function.

As an edible and medicinal fungus, *Ganoderma lucidum* is renowned for its secondary metabolites, ganoderic acids (GAs), which exhibit multiple biological properties, such as anticancer, antioxidant, antiviral, and immunomodulatory properties (19, 20). Our prior research demonstrated that heat stress induces the biosynthesis of polyamines and promotes the conversion of putrescine to spermidine, thereby promoting the accumulation of GAs in *G. lucidum* (21). Subsequent research indicated that heat stress activates the transcription factor Glmyb via phospholipase D-mediated phosphatidic acid to regulate the expression of the *spdS* gene, which encodes spermidine synthase, a key enzyme in the biosynthesis of spermidine, thus controlling spermidine biosynthesis (22). However, as a key stress response factor, the impact of spermidine on the heat tolerance of *G. lucidum* and the underlying regulatory mechanisms are not yet fully understood. In this study, we uncovered that spermidine promoted mitochondrial respiration by promoting the translation of long chain acyl-CoA dehydrogenase (LCAD) and mitochondrial trifunctional protein (HADH), both of which are key enzymes for long-chain fatty acid β-oxidation, thereby improving the heat tolerance of *G. lucidum*. These results indicate that spermidine plays a significant role in metabolic regulation under heat stress and enhances the heat tolerance of *G. lucidum*, contributing to the overall understanding of fungal heat stress tolerance.

## RESULTS

### Spermidine enhances the heat tolerance of *G. lucidum*

**Fig 1:**
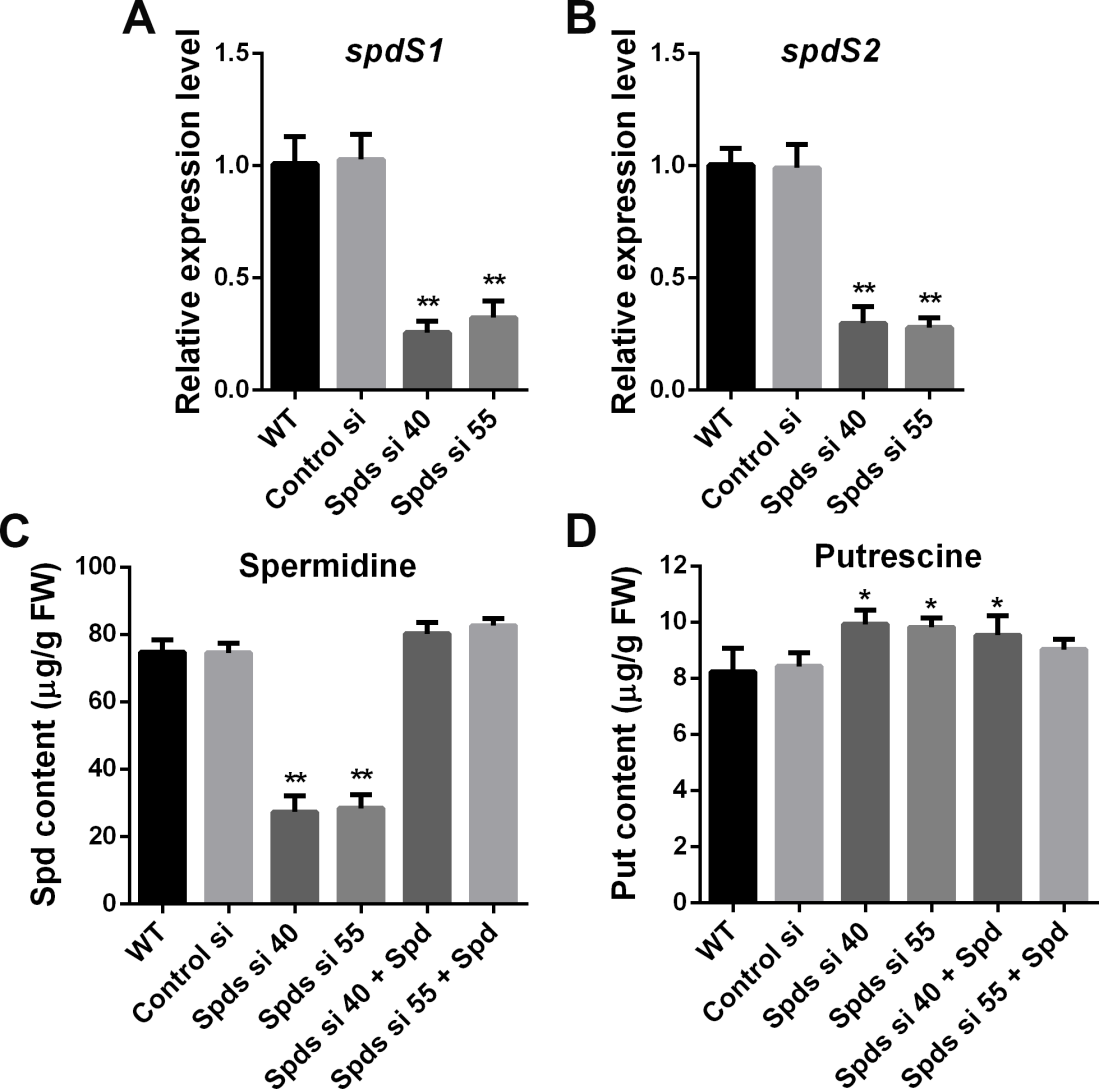
Analysis of *spdS* expression levels and polyamine contents. (**A-B**) The expression levels of *spdS1* and *spdS2* in wild-type (WT), empty vector control (Control si), and *spdS* knockdown strains (Spds si 40 and Spds si 55, originally named GlSpds-kd40 and GlSpds-kd55, respectively, as per Tao et al.) in CYM solid medium. (**C-D**) The contents of spermidine and putrescine in WT, Control si, and *spdS* knockdown strains, as well as in strains supplemented with 1 mM spermidine (Spd) in CYM solid medium. The values presented are the mean ± standard deviation (SD) from three independent experiments (*P < 0.05, **P < 0.01).

**Fig 2:**
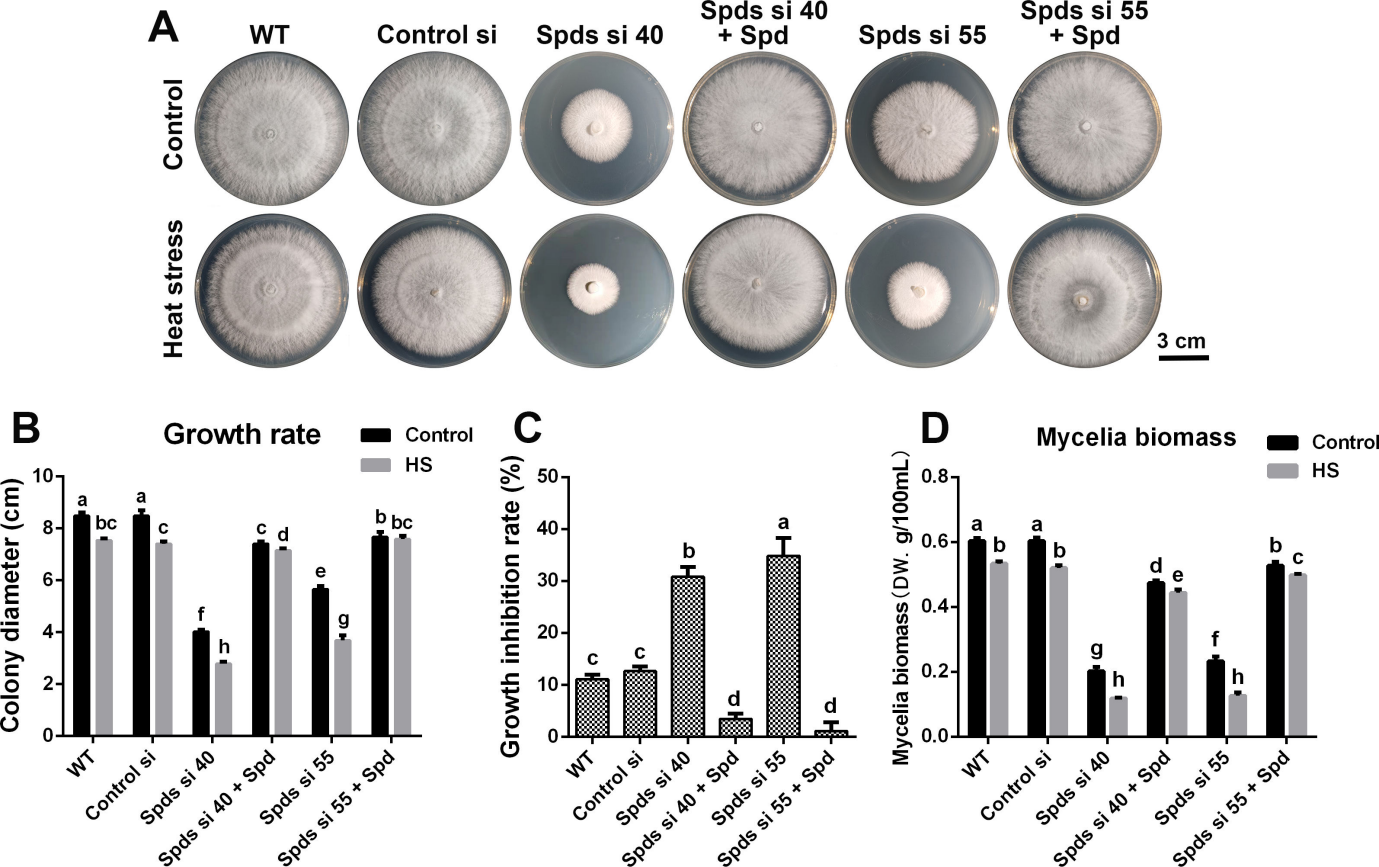
Analysis of the heat tolerance of *G. lucidum* strains. (**A**) The growth status of WT, Control si, *spdS* knockdown strains, and strains supplemented with 1 mM Spd under heat stress (HS) at 42°C for 30 min in CYM solid medium. (**B**) The colony diameter from Panel A. (**C**) The growth inhibition rate of these strains under heat stress (growth inhibition rate = $\frac{d_{control}-d_{HS}}{d_{control}}$). (**D**) The mycelia biomass of WT, Control si, *spdS* knockdown strains, and strains supplemented with 1 mM spd grown under heat stress at 42°C for 30 min in CYM liquid medium. The values presented are the mean ± standard deviation (SD) from three independent experiments. Different letters indicate significant differences between the lines (*P* < 0.05, according to Duncan’s multiple-range test).

Spermidine synthase (SPDS) is a crucial enzyme involved in the biosynthesis of spermidine. Following the knockdown of *spdS*, the level of spermidine was reduced by over 60%, accompanied by a slight increase in putrescine levels in *G. lucidum* (Fig. 1). To investigate the effect of spermidine on heat tolerance in *G. lucidum*, the growth rates were examined in both wild-type (WT) and *spdS* knockdown strains. As shown in Fig. 2A through C, the growth rates across all genotypes were suppressed under heat stress at 42°C for 30 min. However, the growth inhibition rate of the WT was approximately 11.1%, whereas that of *spdS* knockdown strains exceeded 30%, suggesting that *spdS* knockdown results in increased sensitivity to heat stress. In *spdS* knockdown strains, heat stress-induced inhibition was alleviated after supplementation with spermidine. The growth inhibition rates were 3.48% and 1.12%, respectively, in Spds si 40 and Spds si 55 after supplementation with 1 mM spermidine (Fig. 2C). Furthermore, mycelia biomass in liquid medium displayed comparable trends with colony diameter cultivated in solid plate (Fig. 2A vs D). Taken together, these results demonstrate that spermidine significantly enhances the heat tolerance of *G. lucidum*.

### Mitochondrial respiration is essential for spermidine enhanced heat tolerance

**Fig 3:**
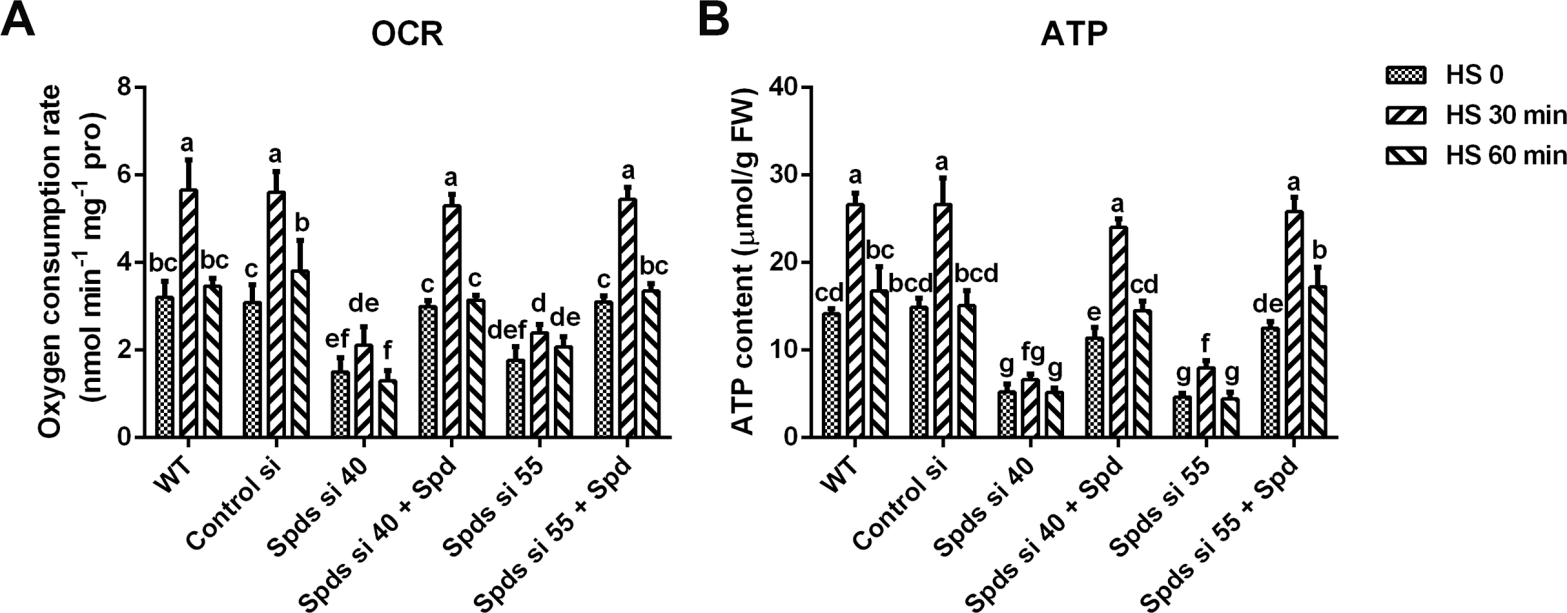
Analysis of the respiratory level of *G. lucidum* strains under heat stress. (**A**) The oxygen consumption rate (OCR) in WT, Control si, *spdS* knockdown strains, and strains supplemented with 1 mM Spd under heat stress grown in CYM liquid medium. (**B**) The ATP levels in these strains are under heat stress. The values presented are the mean ± standard deviation (SD) of three independent experiments. Different letters indicate significant differences between the lines (*P* < 0.05, according to Duncan’s multiple-range test).

**Fig 4:**
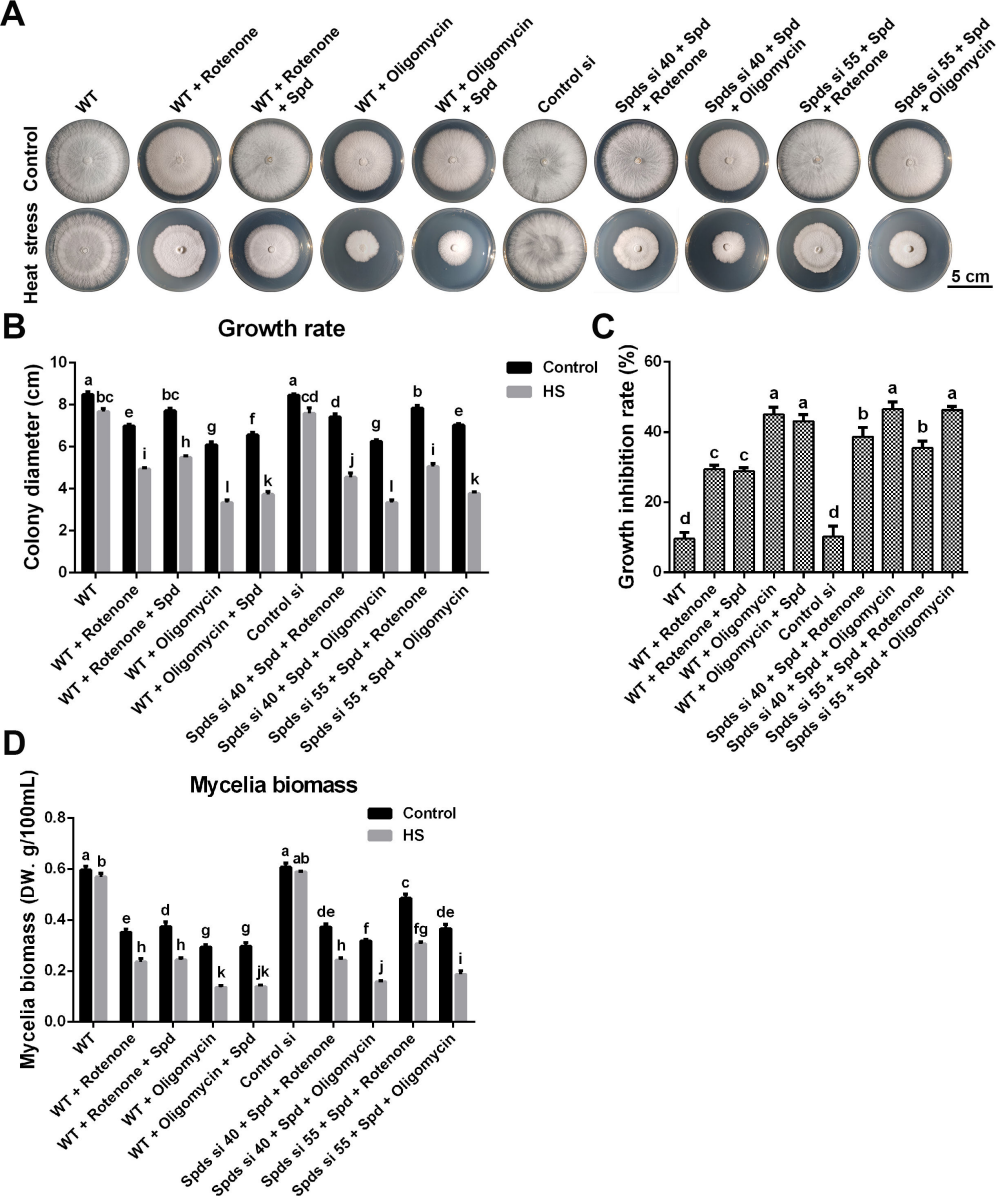
Analysis of the heat tolerance of *G. lucidum* strains after inhibiting mitochondrial respiration. (**A**) The growth status of WT, Control si, *spdS* knockdown strains, and strains treated with rotenone or oligomycin in CYM solid medium. (**B**) The colony diameter from panel A. (**C**) The growth inhibition rate of these strains under heat stress. (**D**) The mycelia biomass of WT, Control si, *spdS* knockdown strains, and strains treated with rotenone or oligomycin grown in CYM liquid medium. The values presented are the mean ± standard deviation (SD) from three independent experiments. Different letters indicate significant differences between the lines (*P* < 0.05, according to Duncan’s multiple-range test).

Mitochondria play a crucial role in cellular homeostasis and are key components of the stress response (14, 15). To ascertain if spermidine enhances the heat tolerance of *G. lucidum* through mitochondrial regulation, mitochondrial respiration was initially examined. Mycelia balls, grown in CYM liquid medium, were utilized to ascertain the oxygen consumption rate (OCR). It was observed that different treatments exerted a negligible impact on mycelial ball morphology, implying that the respiratory state of the entire mycelial balls is likely consistent across various treatments. The results indicated that the OCR and ATP content increased over the course of heat stress and reached a peak at 30 min (Fig. S1). As shown in Fig. 3A, the OCR was 3.2 nmol∙min^−1^∙mg^−1^pro in WT under normal conditions and achieved 5.67 nmol∙min^−1^∙mg^−1^pro after 30 min of heat stress, an increase of nearly 80%, but the increase was suppressed after knocking down the *spdS* gene. In *spdS* knockdown strains, the OCR was decreased by 70%–75% compared with the WT and restored to WT levels after supplementation with 1 mM spermidine under heat stress, and ATP content followed a similar trend to OCR (Fig. 3B). These results suggested that spermidine promotes heat stress-induced mitochondrial respiration. Mitochondria are important sites for ROS production. Assessment of mitochondrial ROS levels and H_2_O_2_ content revealed that heat stress-induced ROS production, which was mitigated by *spdS* gene knockdown (Fig. S2), indicating that spermidine is essential for maintaining mitochondrial function following heat stress. Subsequently, the growth rate under heat stress was examined after inhibition of mitochondrial respiration by rotenone (an inhibitor of mitochondrial complex I) and oligomycin (an inhibitor of ATP synthase). Oxygen consumption and ATP production were inhibited in the WT and *spdS* knockdown strains with or without the addition of spermidine after treatment with rotenone or oligomycin (Fig. S3). Growth rate analysis indicated that the sensitivity of the WT in CYM medium to heat stress was increased after treatment with rotenone or oligomycin, whereas heat resistance did not significantly improve after supplementation with spermidine (Fig. 4A through D). In *spdS* knockdown strains, heat stress-induced inhibition was alleviated after supplementation with spermidine (Fig. 2), but this effect was not observed after treatment with rotenone or oligomycin (Fig. 4A through D). Taken together, these results suggest that mitochondrial respiration is essential for spermidine-enhanced thermotolerance of *G. lucidum*.

### Spermidine promotes mitochondrial respiration by accelerating the TCA cycle and facilitating electron transport

**Fig 5:**
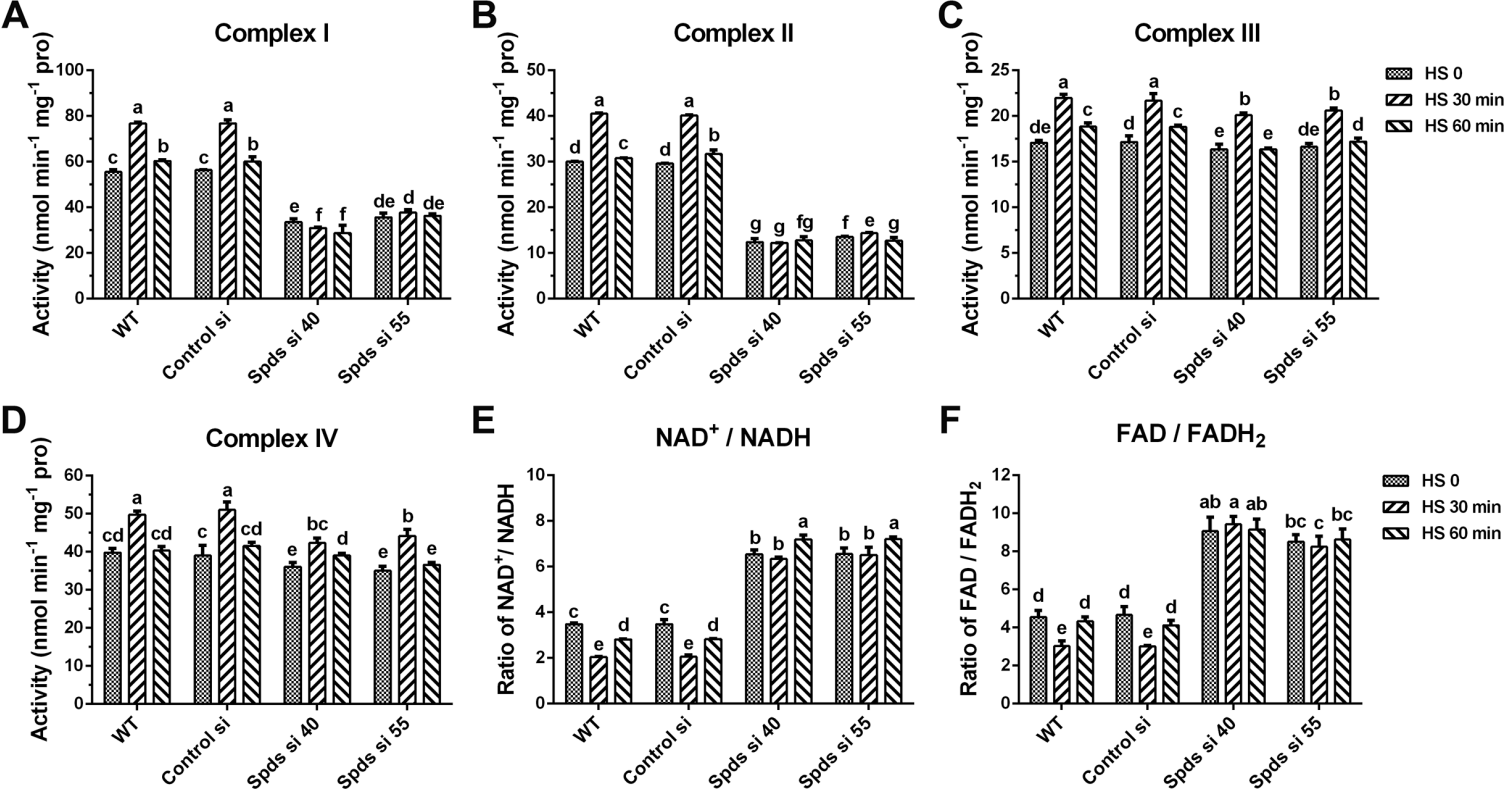
Analysis of mitochondrial complex activities and the ratio of NAD^+^/NADH and FAD/FADH_2_ under heat stress. (**A-D**) Activities of mitochondrial complexes I, II, III, and IV in WT, Control si, and *spdS* knockdown strains under heat stress in CYM solid medium. (**E**) The ratio of mitochondrial NAD^+^/NADH in WT, Control si, and *spdS* knockdown strains under heat stress in CYM solid medium. (**F**) The ratio of intracellular FAD/FADH_2_ in WT, Control si, and *spdS* knockdown strains under heat stress in CYM solid medium. The values presented are the mean ± standard deviation (SD) from three independent experiments. Different letters indicate significant differences between the lines (*P* < 0.05, according to Duncan’s multiple-range test).

**Fig 6:**
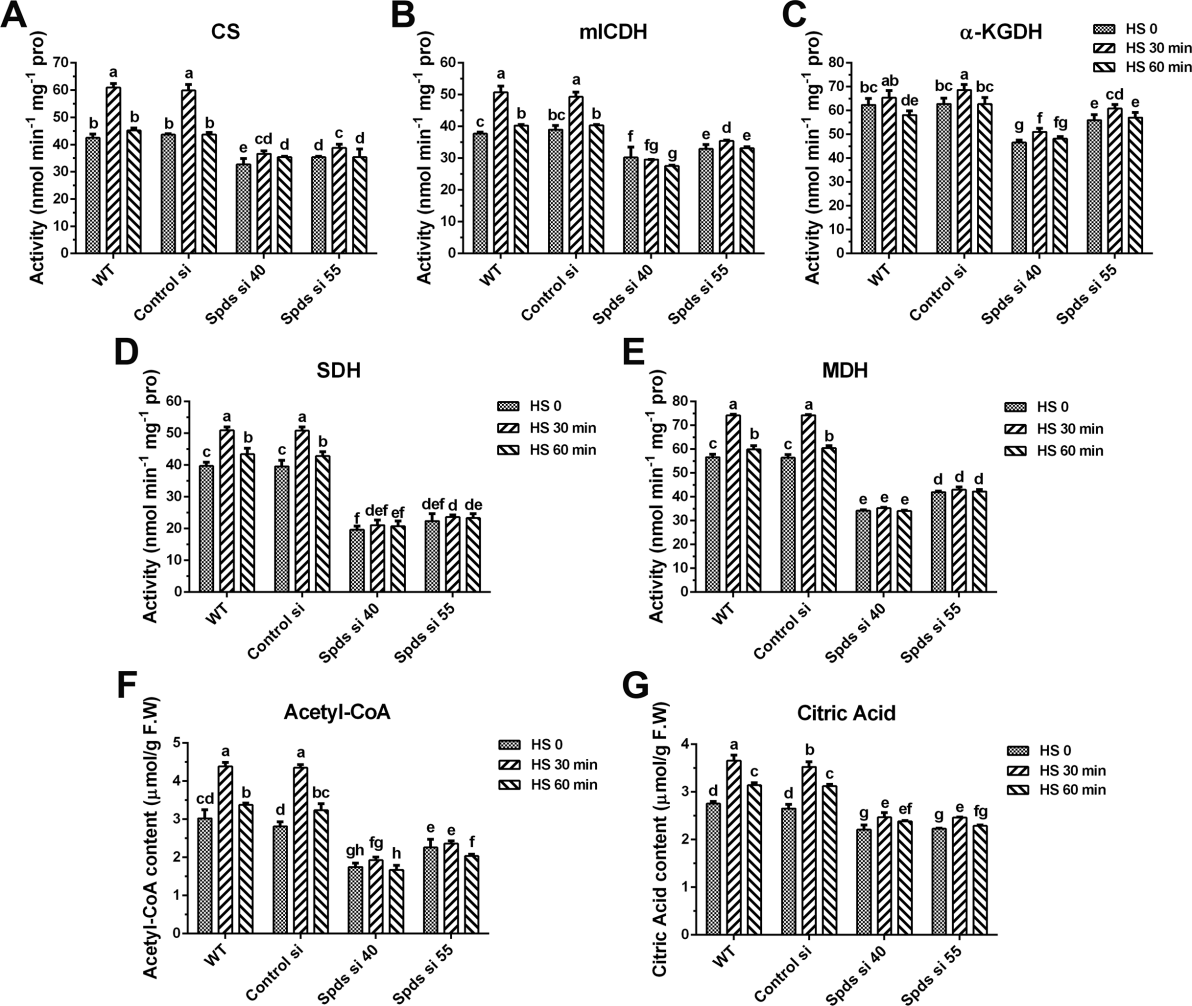
Analysis of key enzyme activities from the TCA cycle and substrate content under heat stress. (**A-E**) Activities of citrate synthase (CS), mitochondrial isocitrate dehydrogenase (mICDH), α-ketoglutarate dehydrogenase (α-KGDH), succinate dehydrogenase (SDH), and malate dehydrogenase (MDH) in WT, Control si, and *spdS* knockdown strains under heat stress in CYM solid medium. (**F**) Acetyl-CoA contents in WT, Control si, and *spdS* knockdown strains under heat stress in CYM solid medium. (**G**) Ctric acid contents in WT, Control si, and *spdS* knockdown strains under heat stress in CYM solid medium. The values presented are the mean ± standard deviation (SD) from three independent experiments. Different letters indicate significant differences between the lines (*P* < 0.05, according to Duncan’s multiple-range test).

To explore the response of mitochondria to heat stress and the regulation of spermidine in mitochondrial respiration, the activities of mitochondrial complexes and ratios of mitochondrial NAD/NADH and intracellular FAD/FADH_2_ were analyzed. As shown in Fig. 5A through D, the activities of complexes I, II, III, and IV were significantly increased after 30 min of heat stress in the WT. However, the activities of complexes I and II were inhibited after knocking down the *spdS* gene, and the increase induced by heat stress was absent (Fig. 5A and B). NADH and FADH_2_ provide electrons for the respiratory chain, and NADH and FADH_2_ accumulate after blocking electron transport (23). Analysis of mitochondrial NAD/NADH and intracellular FAD/FADH_2_ in the WT showed that, after 30 min of heat stress in the WT, the NAD/NADH and FAD/FADH_2_ ratios decreased by 41.2% and 33.5%, respectively, revealing that heat stress promotes the accumulation of NADH and FADH_2_ (Fig. 5E and F). However, the reduction was absent in *spdS* knockdown strains after 30 min of heat stress. Compared with WT without heat stress, the NAD/NADH and FAD/FADH_2_ ratios of *spdS* knockdown strains increased by 2.1-fold and 1.7-fold to 2.0-fold, respectively, revealing that knockdown of *spdS* decreases the levels of NADH and FADH_2_. However, this result was inconsistent with the hypothesis that *spdS* knockdown would induce NADH and FADH_2_ accumulation by inhibiting complexes I and II. Thus, knockdown of *spdS* may also inhibit the production of NADH and FADH_2_. Mitochondrial NADH is produced from the TCA cycle, and FADH_2_ is produced from the TCA cycle and fatty acid β-oxidation. Subsequently, the activities of key enzymes and the levels of key substrates of the TCA cycle were analyzed. As shown in Fig. 6A through E, after 30 min of heat stress, the activities of citrate synthase (CS), mitochondrial isocitrate dehydrogenase (mICDH), malate dehydrogenase (MDH), and succinate dehydrogenase (SDH) in the WT increased by 43.5%, 34.4%, 31.0%, and 28.3%, respectively. In *spdS* knockdown strains, the activities of CS, mICDH, MDH, and SDH were significantly reduced compared with the WT. However, heat stress caused almost no significant increase in the activity of these enzymes after knocking down *spdS* (Fig. 6A, B, D and E). In addition, under heat stress, the activity of α-ketoglutarate dehydrogenase (α-KGDH) did not significantly change in the WT but was suppressed in *spdS* knockdown strains (Fig. 6C). Analysis of key substrate levels revealed that the levels of acetyl-CoA (Ac-CoA) and citric acid (CA) were increased by 30%–40% after 30 min of heat stress in the WT, but their contents decreased by 46.2%–56.1% and 32.5% in *spdS* knockdown strains under heat stress, respectively (Fig. 6F and G). Taken together, these results suggest that mitochondria respond to heat stress and that spermidine promotes mitochondrial respiration by accelerating TCA cycle flux and facilitating electron transport under heat stress.

### Spermidine enhances heat stress-induced fatty acid β-oxidation

**Fig 7:**
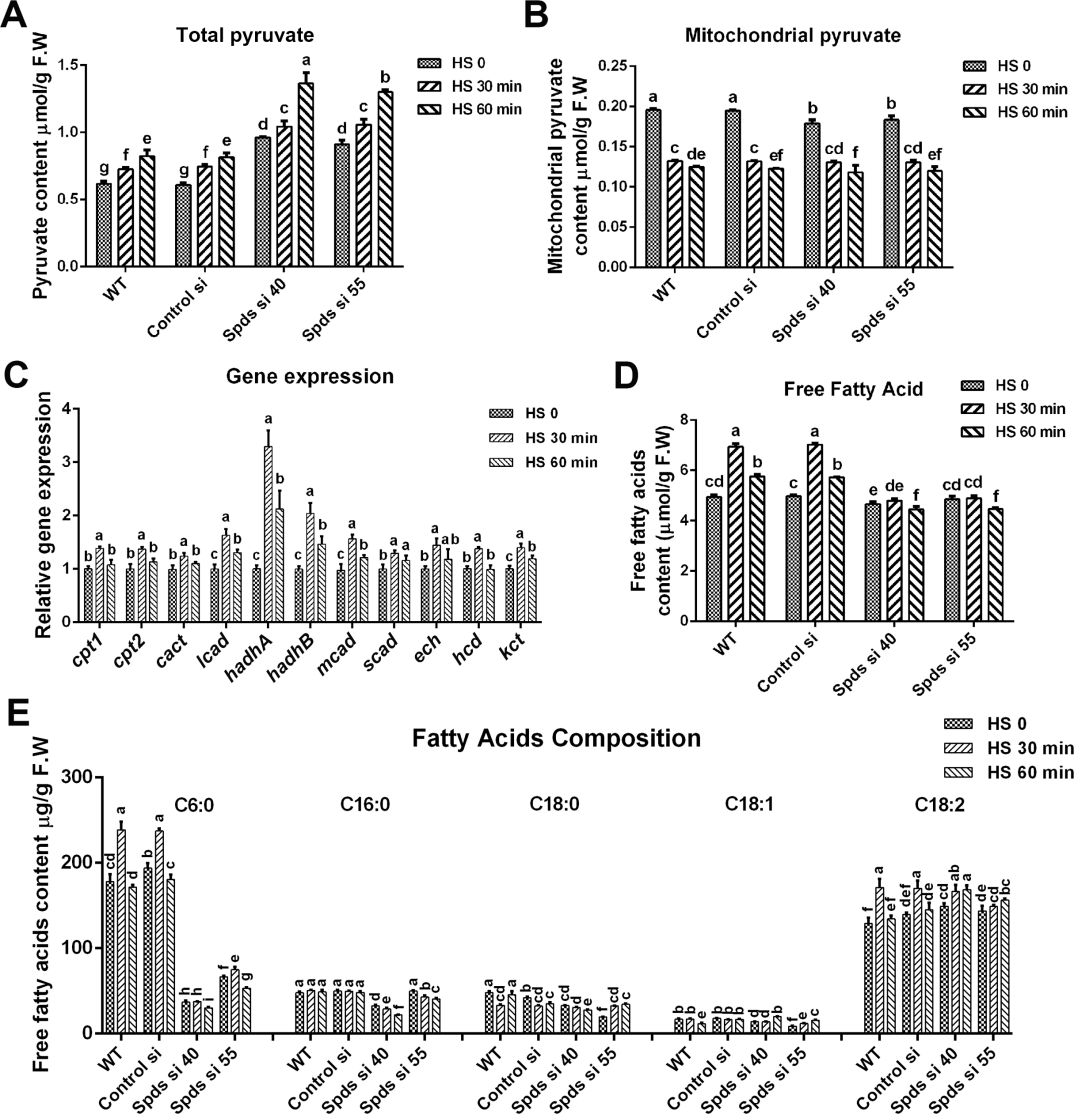
The contents of pyruvate and fatty acids and the expression levels of fatty acid β-oxidation-related genes under heat stress. (**A-B**) The contents of intracellular total pyruvate and mitochondrial pyruvate in WT, Control si, and *spdS* knockdown strains under heat stress in CYM solid medium. (**C**) The expression levels of fatty acid β-oxidation-related genes under heat stress in WT. Fatty acids are first activated to acyl-CoAs in the cytosol and subsequently transported to mitochondria for oxidation with the action of carnitine palmitoyl-transferase (CPT1, CPT2) and carnitine acylcarnitine translocase (CACT). Long-chain acyl-CoAs are metabolized by long-chain acyl-CoA dehydrogenase (LCAD) and mitochondrial trifunctional protein (MTP, also known as HADH, encoded by *hadhA* and *hadhB*), which has hydratase, hydroxyacyl-CoA dehydrogenase, and thiolase activity. Short- and medium-chain acyl-CoAs are metabolized by medium/short-chain acyl-CoA dehydrogenase (M/SCAD), enoyl-CoA hydratase (ECH), β-hydroxyacyl CoA dehydrogenase (HCD), and 3-ketoacyl-CoA thiolase (KCT). (**D**) The contents of free fatty acid in WT, Control si, and *spdS* knockdown strains under heat stress in CYM solid medium. (**E**) The composition and content of free fatty acid in WT, Control si, and *spdS* knockdown strains under heat stress in CYM solid medium. The values presented are the mean ± standard deviation (SD) from three independent experiments. Different letters indicate significant differences between the lines (*P* < 0.05, according to Duncan’s multiple-range test).

The TCA cycle produces the electron carriers NADH and FADH_2_, which are subsequently utilized by the electron transport chain (ETC). Acetyl-CoA, formed from the conversion of pyruvate, serves as the primary substrate that initiates the TCA cycle reactions. Pyruvate is predominantly sourced from the glycolytic pathway. To investigate the glycolysis under heat stress, analyses of phosphofructokinase (PFK), glyceraldehyde 3-phosphate dehydrogenase (GAPDH), and pyruvate kinase (PK) activities were conducted. The activities of PFK, GAPDH, and PK increased significantly and peaked after 60 min of heat stress in the WT, increasing by 5.9-fold, 5.8-fold, and 6.3-fold, respectively (Fig. S4). After 90 min of heat stress, the activities began to decline but remained significantly elevated compared with pre-stress levels (3.0-fold, 3.8-fold, and 2.5-fold, respectively). Upon *spdS* knockdown, the activities of PFK, GAPDH, and PK were markedly elevated compared with WT, irrespective of heat stress. These findings suggest that heat stress stimulates the glycolytic pathway over the long term (at least 90 min) and that glycolysis is further accelerated following *spdS* knockdown, diverging from the TCA cycle response. To ascertain whether the acceleration of mitochondrial respiration under heat stress is pyruvate-driven, total pyruvate and mitochondrial pyruvate levels were detected. As shown in Fig. 7A and B, total pyruvate progressively accumulated under heat stress in the WT, increasing by 33.6% at 60 min of heat stress. Conversely, mitochondrial pyruvate levels diminished by 36.1% after 60 min of heat stress in the WT. Furthermore, total pyruvate levels increased by 56.8% to 66.0% in *spdS* knockdown strains relative to the WT under heat stress, but mitochondrial pyruvate levels remained comparable with those of the WT. These results indicate that pyruvate import into mitochondria is impeded under heat stress, and *spdS* knockdown facilitates pyruvate accumulation but exerts minimal influence on pyruvate import into mitochondria.

Acetyl-CoA, generated from fatty acid β-oxidation, directly feeds into the TCA cycle during aerobic respiration (24), and fatty acid β-oxidation is a primary source of FADH_2_ production. To ascertain if heat stress-induced mitochondrial respiration is mediated by fatty acid β-oxidation, the expression levels of genes related to fatty acid β-oxidation were evaluated. As shown in Fig. 7C, the expression levels of fatty acid β-oxidation-related genes were significantly increased in the WT under heat stress. Furthermore, analysis of free fatty acids content demonstrated that levels of free fatty acids increased by 28.8% in the WT after 30 min of heat stress, but this increase was not observed in *spdS* knockdown strains, which exhibited levels significantly lower than those of the WT (Fig. 7D), exhibiting a pattern consistent with Ac-CoA levels (Fig. 6F). Subsequently, the composition of free fatty acids was analyzed. As shown in Fig. 7E, the concentration of medium-chain fatty acid (C6:0) increased by 33.9% after 30 min of heat stress in the WT and decreased by 68.6%–84.2% following *spdS* knockdown under heat stress. However, the concentration of long-chain fatty acid (C18:2) was marginally elevated in *spdS* knockdown strains. Collectively, these findings indicate that spermidine enhances heat stress-induced fatty acid β-oxidation.

### Spermidine promotes the translation of LCAD and HADH

**Fig 8:**
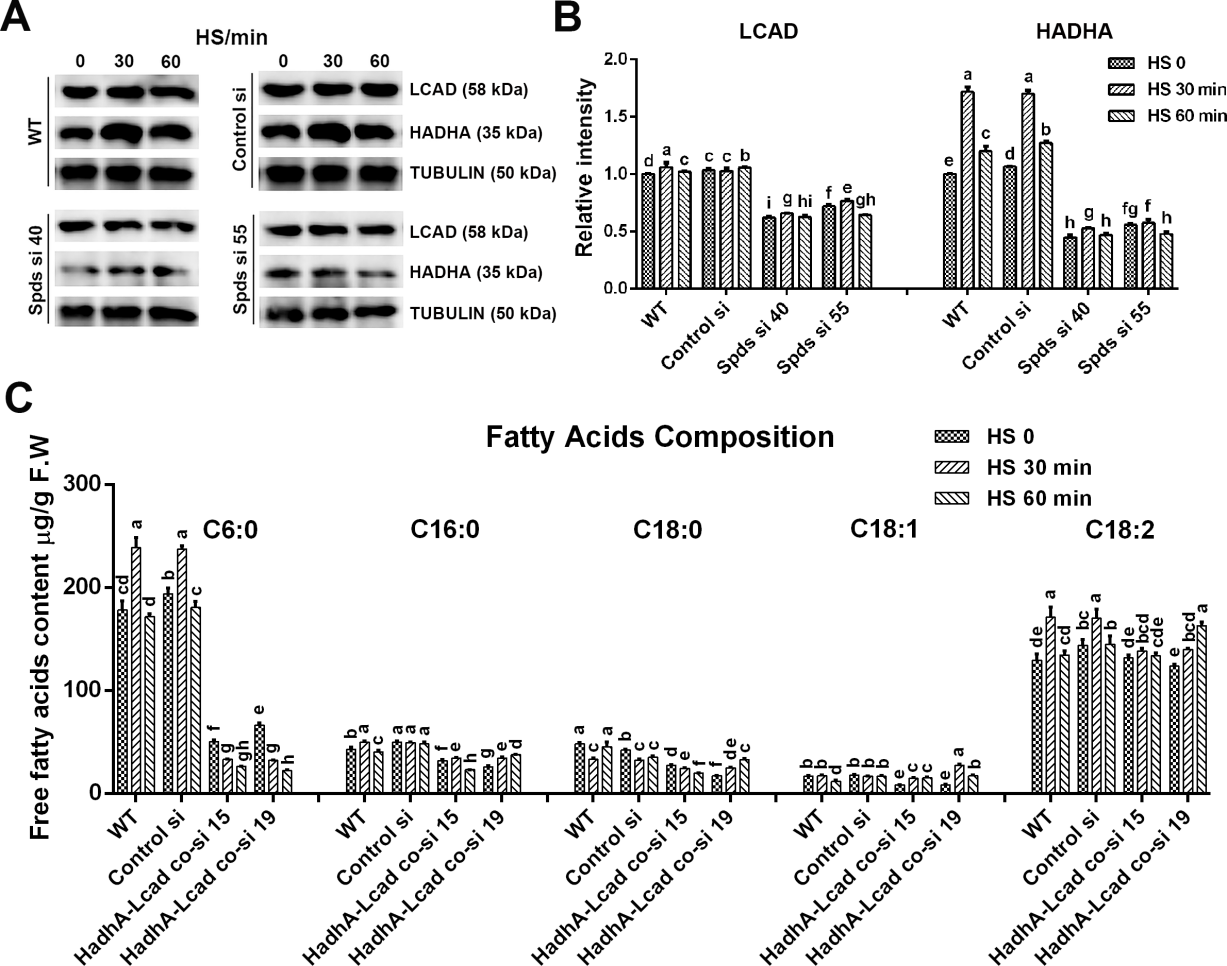
Analysis of the protein levels of LCAD and HADH and the composition and content of fatty acid under heat stress. (**A**) The LCAD and HADH protein levels in WT, Control si, and *spdS* knockdown strains under heat stress in CYM solid medium were detected by Western blot. (**B**) The relative intensity from Panel A. (**C**)The composition and content of free fatty acid in WT, Control si, *lcad,* and *hadhA* co-knockdown strains (HadhA-Lcad co-si 15 and HadhA-Lcad co-si 19) under heat stress in CYM solid medium. The values presented are the mean ± standard deviation (SD) from three independent experiments. Different letters indicate significant differences between the lines (*P* < 0.05, according to Duncan’s multiple-range test).

The inhibition of long-chain fatty acid β-oxidation (e.g., deficiency of CPT, LCAD, or HADH) leads to accumulation of long-chain fatty acids and a decrease of short/medium-chain fatty acid (25). To explore the effect of spermidine on long-chain fatty acid β-oxidation under heat stress, the gene transcriptional levels and polysomal mRNA fractions related to the transport and β-oxidation of long-chain fatty acid were assessed. Results indicated that transcriptional levels of genes related to the transport and β-oxidation of long-chain fatty acid increased in the WT under heat stress, particularly *lcad*, *hadhA,* and *hadhB* (Fig. S5), but were not significantly affected after knocking down *spdS*. However, the polysomal mRNA fractions of *lcad*, *hadhA,* and *hadhB* were significantly inhibited after knocking down the *spdS* gene. Subsequently, protein levels of HADH and LCAD were analyzed. As shown in Fig. 8A and B, HADH increased by 72% in the WT after 30 min of heat stress. However, the increase induced by heat stress was suppressed after knocking down *spdS*, and its protein level was significantly lower than that of the WT. Under heat stress, LCAD levels barely increased in the WT but significantly decreased in *spdS* knockdown strains. These results suggest that spermidine promotes the translation of LCAD and HADH under heat stress mainly by increasing polysomal mRNA fractions of *lcad*, *hadhA* and *hadhB*. Subsequently, *lacd* and *hadhA* gene co-knockdown strains were constructed, and two high-efficiency co-knockdown strains were screened (Fig. S6). Analysis of free fatty acid composition showed that the medium-chain fatty acid (C6:0) level was significantly decreased in *lacd* and *hadhA* co-knockdown strains, and the proportion of long-chain fatty acid (C18:2) was increased (Fig. 8C), which showed similar trends to *spdS* knockdown strains (Fig. 7E). Collectively, spermidine accelerates fatty acid β-oxidation by promoting the translation of LCAD and HADH under heat stress.

### LCAD and HADH promote mitochondrial respiration and thermotolerance of *G. lucidum*

**Fig 9:**
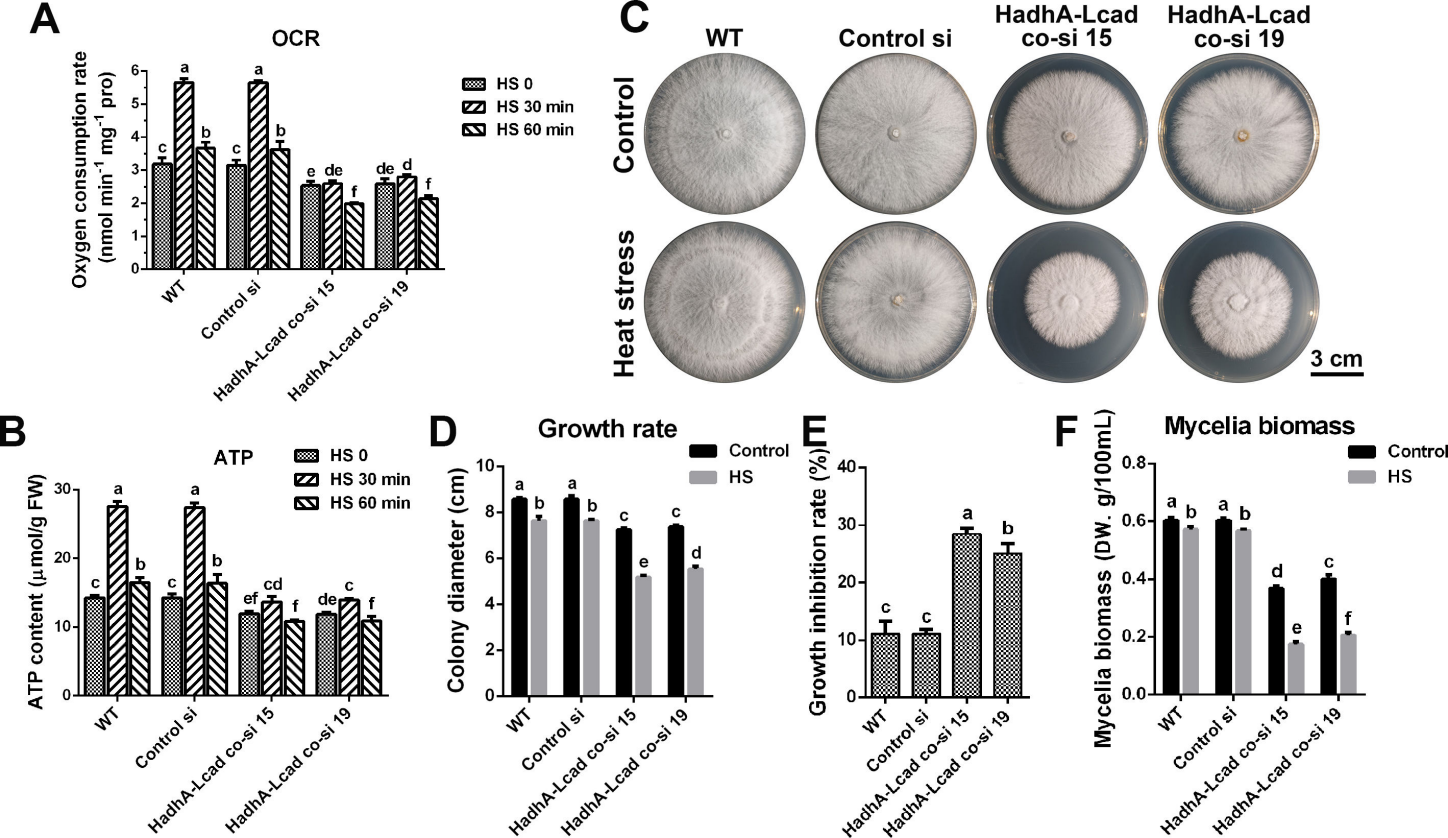
Analysis of respiratory level and heat tolerance of *lcad* and *hadhA* co-knockdown strains. (**A-B**) The oxygen consumption rate and ATP contents in WT, Control si, *lcad* and *hadhA* co-knockdown strains under heat stress grown in CYM liquid medium. (**C**) The growth status of WT, Control si, *lcad,* and *hadhA* co-knockdown strains under heat stress in CYM solid medium. (**D**) The colony diameter from Panel C. (**E**) The growth inhibition rate of these strains under heat stress. (**F**) The mycelia biomass of WT, Control si, *lcad* and *hadhA* co-knockdown strains grown in CYM liquid medium. The values presented are the mean ± standard deviation (SD) from three independent experiments. Different letters indicate significant differences between the lines (*P* < 0.05, according to Duncan’s multiple-range test).

To investigate the function of LCAD and HADH in the heat tolerance of *G. lucidum*, the OCR, ATP levels, and growth rates were analyzed in *lacd* and *hadhA* co-knockdown strains. As shown in Fig. 9A and B, the OCR and ATP levels significantly increased in the WT after 30 min of heat stress, but the increase induced by heat stress was suppressed in *lacd* and *hadhA* co-knockdown strains. Furthermore, the OCR and ATP levels were significantly lower than those of the WT, revealing that knockdown of *lacd* and *hadhA* reduces the heat stress-induced enhancement of mitochondrial respiration. Although the growth of both WT and knockdown strains was inhibited under heat stress, the growth inhibition rate of *lacd* and *hadhA* co-knockdown strains was significantly higher than that of the WT (Fig. 9C through E), revealing that knockdown of *lacd* and *hadhA* results in an increased sensitivity to heat stress. Analysis of mycelia biomass in liquid medium showed similar results (Fig. 9F). Altogether, these results demonstrated that LCAD and HADH promote heat stress-induced mitochondrial respiration and thus enhance the heat tolerance of *G. lucidum*.

## DISCUSSION

High temperature, a common environmental stress, generally exerts negative effects on organisms and has widespread effects on metabolism (26, 27). In plants, heat stress alters the physical state of membranes and affects membrane proteins as well as the membrane structure, promoting the release of phospholipid signals and inducing ROS production and cytosolic Ca^2+^ burst, which alter cellular homeostasis by regulating metabolic processes to adapt to external conditions (28). In our previous studies on *G. lucidum*, we found that heat stress induces oxidative stress and cytosolic Ca^2+^ release, resulting in inhibition of mycelium growth and accumulation of secondary metabolite GAs (29, 30). Spermidine also serves as an important abiotic stress response factor that enhances the tolerance of organisms to various stresses (10). In *G. lucidum*, it was observed that heat stress induces the expression of polyamines biosynthesis-related genes, thus promoting the biosynthesis of polyamines and accelerating the conversion of putrescine to spermidine (21). Subsequent studies have shown that heat stress activates the transcription factor Glmyb via phospholipid signaling to promote the expression of the *spdS* gene, thus accelerating the biosynthesis of spermidine (22). Consequently, research has increasingly focused on the function of spermidine. Spermidine promotes the translation of mitochondrial complex I and II by hypusinating eIF5A, thus enhancing mitochondrial function (18). However, the role of spermidine in heat stress resistance and whether there is a potential connection between the enhancement of mitochondrial function and the heat resistance of *G. lucidum* remains unclear. In this study, we found that spermidine enhances the heat tolerance of *G. lucidum* by promoting mitochondrial function. Further research revealed that spermidine enhances key TCA cycle and ETC enzyme activities and promotes the translation of the key long-chain fatty acid β-oxidation enzymes LCAD and HADH, thereby promoting mitochondrial respiration and ATP production. Our findings reveal a preliminary mechanism by which spermidine is involved in the heat stress-induced mitochondrial stress response and metabolic rearrangement to improve the heat tolerance of *G. lucidum*. This study provides new insights into the understanding of fungal stress response.

Spermidine, an important member of the polyamine family, is an essential organic cation that is present in all living organisms. Plants possess two pathways for polyamine biosynthesis. For instance, putrescine is synthesized by the decarboxylation of ornithine, catalyzed by ornithine decarboxylase (ODC), or indirectly by the decarboxylation of arginine decarboxylase (ADC) (31). Subsequently, putrescine is converted into spermidine by SPDS. Decarboxylated S-adenosylmethionine produced by S-adenosylmethionine decarboxylase (SAMDC) provides the amino group for spermidine synthesis. In general, decarboxylation of ornithine by ODC is accepted as the only way to produce putrescine in fungi (32). In *G. lucidum*, the arginine decarboxylase pathway has not been identified. In fungi, ODC is a key enzyme in putrescine biosynthesis. In addition, SAMS (S-adenosylmethionine synthetase) and SAMDC are also two crucial enzymes in polyamine biosynthesis. They play a significant role in polyamine biosynthesis along with ODC (33). The conversion of putrescine to spermidine by SPDS is the only way of spermidine biosynthesis found so far (32). In *G. lucidum*, it was observed that the growth was significantly inhibited after knocking down *spdS* in CYM medium. To eliminate the influence of residual polyamines from medium on the growth, a polyamine-free medium was used. The growth rate was significantly inhibited under heat stress after knocking down *spdS*, markedly exceeding the inhibition rate observed in the WT (Fig. S7). These results were consistent with the growth trend noted in solid CYM medium (Fig. 2A through C), suggesting that spermidine plays a crucial role in the growth of *G. lucidum* and that the sensitivity of *G. lucidum* to heat stress increased after *spdS* knockdown. In CYM medium, even with a small amount of polyamines present, the growth of *spdS* knockdown strains was severely constrained due to defects in spermidine synthesis, leading to a significantly reduced growth rate compared with the WT (Fig. 2). Furthermore, *G. lucidum* persists in survival after knocking down *spdS*, likely due to residual gene activity, in addition to the low concentration of polyamines in the CYM medium. In plants, downregulating *samdc* via RNA interference strategy decreases biomass production of tobacco under salt stress, and residual SAMDC activity still maintains the production of a certain amount of polyamines (34). Downregulating *odc* in tobacco results in reduced polyamines production, inhibiting growth and flowering, but the plant can still survive (35). In *G. lucidum*, analysis of gene expression levels showed that the expression level of *spdS1* and *spdS2* decreased significantly, but there was still a small amount of gene activity. Analysis of polyamine levels also showed that spermidine content was significantly reduced (by more than 60%) after knocking down *spdS*, but still, a certain amount of spermidine remained (Fig. 1). This evidence indicates that residual gene activity maintains the production of a certain amount of spermidine, which plays a crucial role in sustaining the survival of *G. lucidum*. However, the deficiency of spermidine significantly impedes its growth.

Environmental stress induces alterations in intracellular metabolism to enhance the adaptability of the organism. In fungi, heat stress promotes glycolysis and inhibits the entry of pyruvate into mitochondria, resulting in the accumulation of intracellular pyruvate. The accumulated pyruvate scavenges heat-induced ROS, efficiently reduces protein carbonylation, and stabilizes mitochondrial membrane potential in *M. robertsii* (8). Additionally, energy homeostasis effectively enhances heat tolerance in plants. In *Arabidopsis thaliana*, knockdown of mitochondrial ATP synthase subunit D results in a reduction in ATP production and heat tolerance (16). Mitochondria are the center of energy metabolism, play a crucial role in cellular homeostasis, and are key components of the stress response (14, 15). Hu et al. found that short-term heat stress (30 min) enhances mitochondrial oxidative phosphorylation in *G. lucidum*. With the extension of heat stress time, the metabolic flow turns to anaerobic glycolysis mediated by Glsnf1, thus enhancing the adaptation to heat stress (36). Low oxygen saturation at higher temperatures may also be a factor in altered respiration. In *Aspergillus terreus*, oxygen saturation affects metabolism and alters respiration during submerged fermentation (37). In general, the mycelia on the surface should be growing aerobically, and aerobic respiration of internal mycelia is inhibited to a certain extent during liquid culture. Considering that our treatment did not change the morphology of mycelial balls, it is likely that the respiratory state of the whole mycelial balls is similar between different treatments. We observed a rapid enhancement in mitochondrial respiratory under heat stress, with the activities of several key enzymes of TCA cycle and ETC peaking at 30 min, followed by a decline in respiratory and a return to pre-heat stress levels by approximately 60 min. This pattern aligns with the variation in spermidine content under heat stress (22), further implying a regulatory role for spermidine in mitochondrial function. In addition, there were likely other mechanisms involved in the transformation of respiration after 30 min of heat stress. For example, Glsnf1-mediated aerobic respiration shifts to anaerobic glycolysis after 30 min of heat stress, resulting in decreased oxygen consumption and ATP production (36). In this study, spermidine promoted mitochondrial respiration to maintain energy homeostasis, playing an important role in the heat tolerance of *G. lucidum*. However, how energy homeostasis regulates heat tolerance remains unclear and requires further investigation.

Pyruvate produced by glycolysis is the main substrate that drives mitochondrial respiration. Heat stress-induced expression of glycolysis-related genes in *G. lucidum* (36). Our findings revealed that heat stress promoted the activities of the key enzymes in glycolysis, thus accelerating pyruvate production. Pyruvate is transported into the mitochondria by the mitochondrial pyruvate carrier (Mpc). In *M. robertsii*, *mpc* gene expression is inhibited, but glycolysis-related genes expression increased under heat stress, resulting in pyruvate accumulation (8). The accumulated pyruvate defends against heat stress by scavenging heat-induced ROS, reducing protein carbonylation and stabilizing mitochondrial membrane potential (8). In this study, significant pyruvate accumulation was also observed under heat stress, but mitochondrial pyruvate significantly decreased, revealing that heat stress inhibits pyruvate entry into mitochondria, leading to pyruvate accumulation in *G. lucidum*, consistent with the report by Zhang et al. (8). The accumulated pyruvate likely acts as a ROS scavenger or a protector against protein carbonylation rather than entering the mitochondria as a driver of mitochondrial respiration. In animals, there are several pathways to maintain the TCA cycle and cell survival during impaired mitochondrial pyruvate transport, such as glutamine oxidation and fatty acid β-oxidation (24, 38). In this study, findings indicated that fatty acid β-oxidation is essential for heat stress-induced enhancement of mitochondrial respiration, as heat stress inhibits pyruvate entry into mitochondria. Other pathways may also be involved in maintaining the TCA cycle, requiring further investigation.

In this study, we observed that the activities of the key enzymes of the mitochondrial TCA cycle and mitochondrial complexes I and II were significantly diminished after knocking down *spdS* gene. In addition, the content of short-chain fatty acids was significantly decreased, accompanied by an increase in the proportion of long-chain fatty acids, and the protein levels of HADH and LCAD, implicated in the β-oxidation of long-chain fatty acids, were also significantly decreased after knocking down *spdS* gene. We consider that spermidine regulates mitochondrial respiration under heat stress via two potential pathways: upregulating key TCA cycle and ETC enzyme activities, and facilitating long-chain fatty acid β-oxidation. Spermidine may also affect the function of enzymes related to the TCA cycle, ETC, and fatty acid β-oxidation in multiple ways. First, hypusination of eIF5A is a key mechanism by which spermidine regulates mitochondrial function. Our previous work has shown that spermidine promotes the translation of mitochondrial complexes I and II and subsequently enhances their activities by hypusinating eIF5A (18). In animals, eIF5A is closely related to mitochondrial function (39, 40). In this study, we found that spermidine promotes the translation of LCAD and HADH to promote long-chain fatty acid β-oxidation, improving the heat tolerance of *G. lucidum*. Second, as a polycation, spermidine binds directly to negatively charged proteins. In mice, spermidine activates HADH by directly binding to the protein, enhancing long-chain fatty acid β-oxidation and improving antitumor immunity (41). However, further investigation is required to ascertain whether spermidine regulates mitochondrial function through multiple cooperative pathways in *G. lucidum*. Mitochondria serve as crucial sites for cellular energy metabolism and are intricately linked to diverse biological processes via substance metabolism (14). Whether/how spermidine modulates stress tolerance in organisms through the regulation of mitochondrial function also merits further exploration.

**Fig 10:**
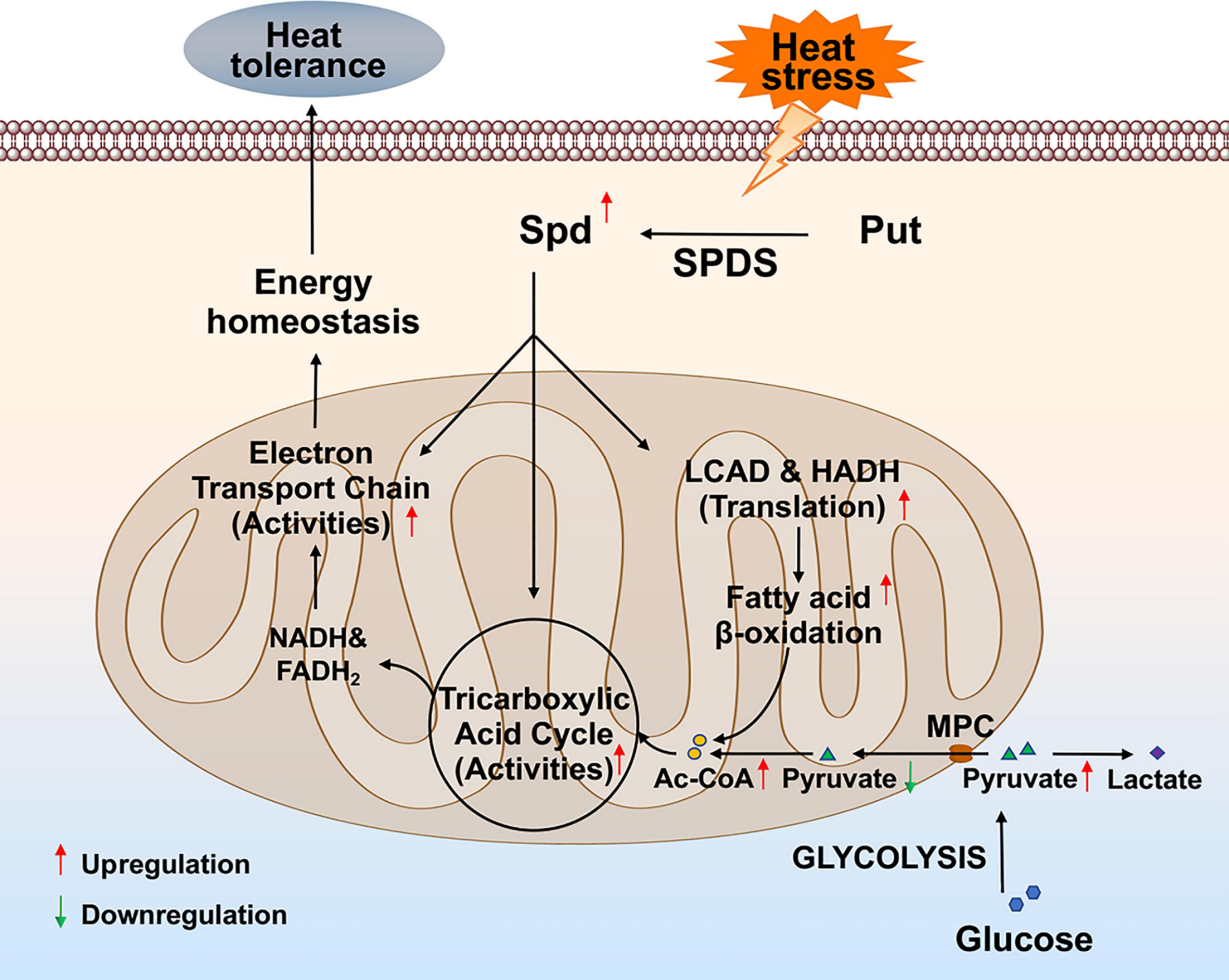
Schematic depicting a model by which spermidine enhances the heat tolerance of *G. lucidum*. Spermidine enhances key TCA cycle and ETC enzyme activities and facilitates heat stress-induced fatty acid β-oxidation by promoting the translation of LCAD and HADH, thereby accelerating mitochondrial respiration and enhancing the heat tolerance of *G. lucidum*.

Polyamines, essential organic cations, are present in all living organisms. Besides being an organic small molecule, polyamines also serve as an inorganic cation. Polyamines are essential for maintaining cellular ion homeostasis. In mammals, polyamines have direct effects on several ion channels or receptors, involving in the regulation of Ca^2+^, Na^+^, and K^+^ homeostasis (42). In *Salmonella*, coordinated regulation of polyamines and Mg^2+^ maintains overall cation homeostasis (43). Notably, many environmental and intracellular factors affect Mg^2+^ homeostasis, and Mg^2+^ enhances the tolerance to multiple abiotic stress (44). The potential relationship between spermidine-mediated enhanced heat resistance of *G. lucidum* and Mg^2+^ remains unclear. In this study, mitochondrial respiration plays a crucial role in spermidine-enhanced heat tolerance of *G. lucidum*. Subsequent analysis revealed that spermidine enhances key TCA cycle and ETC enzyme activities and is involved in heat stress-induced fatty acid β-oxidation by promoting the translation of LCAD and HADH. Based on these results, a potential cellular cascade for spermidine-enhanced heat tolerance by promoting mitochondrial respiration was proposed (Fig. 10). Our findings reveal a preliminary mechanism by which spermidine enhances the heat tolerance of *G. lucidum*, providing insights into the role of polyamines under abiotic stress in fungi.

## MATERIALS AND METHODS

### Strains and culture conditions

*G. lucidum* strain ACCC53264 from the Agricultural Culture Collection of China is designated as the wild type (WT). The *spdS* gene knockdown strains were established in our previous studies, and an empty vector was used as control (Control si) (18, 21). These strains were grown in CYM medium (1% maltose, 2% glucose, 0.2% yeast extract powder, 0.2% tryptone, 0.05% MgSO_4_∙7H_2_O, and 0.46% KH_2_PO_4_) at 28°C for 5–7 days, followed by heat stress treatment at 42°C according to the system established by Zhang et al. (29).

### Analysis of the heat tolerance of *G. lucidum*

The strains were grown in CYM solid medium at 28°C for 5 days and subsequently heat treated at 42°C for 30–60 min, and then, the strains were recultivated at 28°C for 2 days. The colony morphology was observed, and the colony diameter was measured. The growth inhibition ratio was calculated according to the following formula: growth inhibition rate = $\frac{d_{control}-d_{HS}}{d_{control}}$.

### Detection of oxygen consumption rate

The oxygen consumption rate (OCR) was assessed according to the previous description, with slight modifications (44). Fresh mycelia balls, grown in CYM liquid medium at 28°C, were then transferred into a reaction chamber with 1mL reaction medium (0.5 mL sterile water + 0.5 mL CYM liquid medium), and a Clark-type oxygen electrode (Hansatech, UK) was used to monitor the oxygen concentration at 28°C. For heat stress, the mycelia balls were grown in CYM liquid medium at 28°C for 7 days and subsequently heat treated at 42°C for 30–60 min. Immediately afterward, mycelia balls were transferred into a reaction chamber, and oxygen concentration was monitored with a Clark-type oxygen electrode at 42°C. The OCR was calculated based on oxygen consumption and mycelial protein content.

### Metabolite analysis

The strains were grown in CYM solid medium at 28°C for 5 days and subsequently heat treated at 42°C for 30–60 min, and then, the strains were recultivated at 28°C for 2 days. The mycelia were collected for metabolite analysis. The ATP levels were detected with an ATP content assay kit (Solarbio, China). Briefly, 20 mg of fresh mycelia were homogenized with 200 µL lysis buffer on the ice. After centrifugation, the supernatant was collected for ATP content assay with a multi-mode microplate reader SpectraMax iD5 (Molecular Devices, USA) according to the manufacturer’s protocol. Similarly, the contents of acetyl-CoA and citric acid were detected with an acetyl-coenzyme A assay kit (Sigma-Aldrich, USA) and a citric acid content assay kit (Solarbio, China) according to the manufacturer’s protocol. The mitochondria were isolated from 200 mg of fresh mycelia according to a previous description (45), and the mitochondrial nicotinamide adenine dinucleotide NAD^+^/NADH levels were detected with an NAD^+^/NADH assay kit (Beyotime, China). Flavin adenine dinucleotide (FAD) was detected with a FAD assay kit (Abcam, UK). Reduced flavin adenine dinucleotide (FADH_2_) was detected with a FADH_2_ ELISA kit (Affandi, China).

Pyruvate was extracted and determined according to a previous description (46). Briefly, 200 mg of fresh mycelia were ground with liquid nitrogen and subsequently ultrasonicated in 2 mL of 80% ethanol for 10 min at 20% power. After centrifugation, the supernatant containing total pyruvate was detected with UPLC (Agilent Technologies, USA). For the detection of mitochondrial pyruvate, the mitochondria were isolated from 200 mg of fresh mycelia and resuspended in 2 mL of 80% ethanol. Subsequently, the resuspended mitochondria were ultrasonicated and centrifuged to obtain a supernatant containing mitochondrial pyruvate. The supernatant was detected with UPLC.

### Fatty acid level and composition analysis

The free fatty acid was extracted and detected with a free fatty acid content assay kit (Solarbio, China) according to the manufacturer’s protocol. The profile analysis of fatty acids was performed by gas chromatography (GC) according to a previous description, with slight modifications (47, 48). Briefly, 200 mg of fresh mycelia were ground with liquid nitrogen and collected in a 10 mL centrifuge tube. Subsequently, 4 mL of CHCl_3_-CH_3_OH solution (1:1, vol/vol) and 1 mL of ultrapure water were added into the tube and vortexed for 90 seconds. After centrifugation, the lower organic phase was collected in a new tube, and the upper water phase was re-extracted with 1 mL of chloroform. The combined organic phases were evaporated under a stream of nitrogen to obtain a crude fatty acid extract.

The crude extract was dissolved in 2 mL of 2% sulfuric acid-methanol solution and incubated at 70°C for 50 min. Subsequently, 2 mL of ultrapure water was added to the mixture, extracted with 2 mL of n-hexane, and dried with an appropriate amount of anhydrous sodium sulfate to obtain a fatty acid methyl ester crude solution. The sample solution was detected by GC (Thermo Fisher, TRACE GC Ultra, USA) with a DM-2560 chromatographic column and flame ionization detector (FID), and N_2_ was used as the carrier gas. The 37-component fatty acid methyl ester mix (Supelco CRM47885, USA) was used as the standard.

### Enzymatic activity assay

The strains were grown in CYM solid medium at 28°C for 5 days and subsequently heat treated at 42°C for 30–60 min, and then, the strains were recultivated at 28°C for 2 days. The mycelia were collected for enzymatic activity assay. The activity of citrate synthase (CS) was detected with a CS activity assay kit (Solarbio, China). Briefly, 100 mg of fresh mycelia were homogenized with 1,000 µL extraction buffer on the ice. After centrifugation at 11,000 × *g*, the supernatant was collected for CS activity assay with SpectraMax iD5 according to the manufacturer’s protocol. Similarly, mitochondrial isocitrate dehydrogenase (mICDH), α-ketoglutarate dehydrogenase (α-KGDH), malate dehydrogenase (MDH), succinate dehydrogenase (SDH), phosphofructokinase (PFK), glyceraldehyde 3-phosphate dehydrogenase (GAPDH) and pyruvate kinase (PK) were detected with mICDH activity assay kit (Solarbio, China), α-KGDH activity assay kit (Solarbio, China), MDH activity assay kit (Solarbio, China), SDH activity assay kit (Solarbio, China), PFK activity assay kit (Solarbio, China), GAPDH activity assay kit (Solarbio, China), and PK activity assay kit (Solarbio, China), respectively, according to the manufacturer’s protocol.

The activities of mitochondrial complexes were detected with mitochondrial complex activity assay kits (Solarbio, China), referring to the method adopted by Liu et al. (45). Briefly, 100 mg of fresh mycelia were homogenized with 1,000 µL extraction buffer on the ice. After centrifugation at 600 × *g*, the supernatant was collected and subsequently centrifuged at 11,000 × *g* for 15 min. The pellet was resuspended with 400 µL extraction buffer and crushed with ultrasonication. Subsequently, the assay was performed with SpectraMax iD5 according to the manufacturer’s protocols. Mitochondrial complex I catalyzes the dehydrogenation of NADH to NAD^+^. Activity of complex I was calculated according to the oxidation rate of NADH determined at 340 nm. Accordingly, mitochondrial complex II catalyzes the oxidation of succinic acid to fumaric acid, accompanied by the reduction of FAD to FADH_2_. FADH_2_ further reduces oxidized coenzyme Q (CoQ) to produce reduced CoQ. The reduced CoQ could further reduce 2,6-Dichlorophenolindophenol (DPIP). Activity of complex II was calculated according to the reduction rate of DPIP determined at 605 nm. Mitochondrial complex III transfers electrons from reduced CoQ to cytochrome c to produce reduced cytochrome c. Distinct from oxidized cytochrome c, reduced cytochrome c exhibits characteristic absorption at 550 nm. Activity of complex III was calculated according to the increase rate of reduced cytochrome c determined at 550 nm. Mitochondrial complex IV catalyzes the oxidation of reduced cytochrome c to oxidized cytochrome c. Activity of complex IV was calculated according to the decrease rate of reduced cytochrome c determined at 550 nm. The activities of mitochondrial complex I, II, III, and IV were detected with mitochondrial complex I, II, III, and IV activity assay kits, respectively, according to the corresponding manufacturer’s protocols.

### Gene expression and polysomal fraction analysis

The analysis of gene expression and polysomal fraction was performed according to a previous description (18). Total RNA was isolated from 50 mg of fresh mycelia with 1mL of RNAiso Plus (TaKaRa) and reverse transcribed to cDNA for gene expression analysis with a PrimeScript RT reagent kit (TaKaRa). The relative gene expression levels were determined according to the 2^−△△Ct^ method (49). The ribosome was extracted from 200 mg of fresh mycelia with 2 mL of ribosome extraction buffer (50 mM pH 7.5 Tris-HCl, 400 mM KCl, 30 mM MgCl_2_, 5 mM dithiothreitol, 100 mg/mL cycloheximide, 100 mg/mL chloramphenicol, and 1% Triton X-100). After centrifugation, the supernatant was obtained, and 200 µL of 20% Triton X-100 was added. Subsequently, the mixture was centrifuged, and the supernatant was layered onto 10 mL of a 5%–50% linear sucrose gradient and centrifuged at 170,000 × *g* for 2.5 h at 4°C. The polysome fractions were collected and precipitated in 2 volumes of ice-chilled ethanol at 4°C for 12 h. After centrifugation, polysomal mRNA was obtained. Subsequently, the mRNA was reverse transcribed to cDNA for polysomal fraction analysis with a PrimeScript RT reagent kit (TaKaRa), and the data were also evaluated according to the 2^−△△Ct^ method (49). Three independent cDNA samples were prepared, with each sample tested in triplicate. Duncan’s multiple-range test was used to analyze differences among multiple data sets (Primers are listed in Table 1).

**TABLE 1:** Primers used for gene expression and polysomal fraction analysis

Primers	Sequences (5’ → 3’)	Descriptions
RT-Gl23438-F	GTGTCTGATGCCGACCAAAT	For *cpt1* expression and polysomal fraction analysis
RT-Gl23438-R	ACGACCTCCAAGCACGACT	
RT-Gl23796-F	AGGCGGTCTTGAGTTTCCG	For *cpt2* expression and polysomal fraction analysis
RT-Gl23796-R	CGTGGTTGTGAGTTTGAGGG	
RT-Gl19117-F	CTCCTGCTACCGTTCCCTCTT	For *cact* expression and polysomal fraction analysis
RT-Gl19117-R	ATGGTTTGCTGGTGGATCTTG	
RT-Gl24433-R1-F	GTCGAGCCGCTGTCTTCG	For *hadhA* expression and polysomal fraction analysis
RT-Gl24433-R1-R	GGCAGCCTGTGCGCGGTG	
RT-Gl24433-R2-F	CCTTCATCAACGAGGCTATT	For *hadhB* expression and polysomal fraction analysis
RT-Gl24433-R2-R	CATCCACCATCCGCTCTA	
RT-Gl25839-F	CTCCTCGGTGGCGTCATT	For *lcad* expression and polysomal fraction analysis
RT-Gl25839-R	GGACCTCCTGGACGATACG	
RT-Gl26076-F	CAGAAGCTGGAGGACGAATC	For *mcad* expression level analysis
RT-Gl26076-R	GAGGACTGCCTGAGCGAAT	
RT-Gl26761-F	GTGGCTACCTAAACTCGCAGAG	For *scad* expression level analysis
RT-Gl26761-R	AGTGGTCGCCGTCCTTGA	
RT-Gl20416-F	CCCAGCAGGCACATGAAA	For *ech* expression level analysis
RT-Gl20416-R	CGTGAGCGAGGAAACCAAA	
RT-Gl26059-F	TCGCCGAGTCCTCAAAGG	For *hcd* expression level analysis
RT-Gl26059-R	ACCCAGGCGTGCGTAAAG	
RT-Gl23502-F	TGGAATGATCGACGTTGGTATT	For *kct* expression level analysis
RT-Gl23502-R	TCCTCGGACTCCTGGTTGG	
RT-GL25326-F	CGTCGTCTTCGGCGAGGTT	Cyclophilin gene *cyp* as a reference
RT-GL25326-R	TGCTCCGTAGCGGTGGTC	

### Western blotting assay

The western blotting assay was performed according to a previous description (18). Twenty-five micrograms of sample protein were separated in a 12% SDS-PAGE gel and transferred to a polyvinylidene difluoride membrane (Bio-Rad). Subsequently, the polyvinylidene difluoride membrane was incubated with the primary antibody (anti-eIF5A [1:2,000, rabbit; BBI Solutions], anti-hypusine [1:2,000, rabbit; Millipore], anti-LCAD [1:2,000, rabbit; CMCTAG], anti-HADHA [1:2,000, rabbit; CMCTAG], or anti-β-tubulin [1:2,000, mouse; CMCTAG]). Next, the polyvinylidene difluoride membrane was incubated with a secondary antibody (goat anti-rabbit IgG or goat anti-mouse IgG [1:5,000, HRP-conjugated]) and subsequently observed with an ECL Western blot detection system (Amersham Bioscience). A quantitative western blot analysis was performed according to a previous description, with slight modifications (50). Briefly, the gray bands were inverted to bright bands using ImageJ software. Subsequently, the area of bright bands was selected for intensity analysis. The intensity value of the target protein was normalized relative to a reference protein.

### Establishment of gene knockdown strains

The RNA interference (RNAi) knockdown vector and *G. lucidum* gene knockdown strains were constructed according to a previous description (51). The long-chain acyl-CoA dehydrogenase gene *lcad* and mitochondrial trifunctional protein (3-hydroxyacyl-CoA dehydrogenase) alpha subunit gene *hadhA* were amplified by PCR using *G. lucidum* cDNA as a template and primers Lcad-F/R and HadhA-F/R (listed in Table 2), respectively. Subsequently, *lcad* and *hadhA* were cloned into an RNA interference vector and transferred into *G. lucidum* mediated by *Agrobacterium tumefaciens* (52). Two independent knockdown strains with high efficiency for each gene were selected and used for subsequent experiments. (The key abbreviations used in this paper are presented in Table 3)

**TABLE 2:** Primers used for vector construction^*a*^

Primers	Sequences (5’ → 3’)	Gene accessions and descriptions
HadhA-F HadhA-R	ACTAGTCCACGAACACGTCTTCCA CATCCACCATCCGCTCTA	OM909012; to establish the *hadhA* and *lcad* co-knockdown vector
Lcad-F Lcad-R	CCCGCTATGCGTGGTTAT GGTACCTCAACTGGCACCTTGACGT	OM909013; to establish the *lcad* and *hadhA* co-knockdown vector
HadhA-28a-F	GGATCCATGTCGAAGGAGCTGAAGA	OM909012; to establish the HADHA protein expression vector
HadhA-28a-R	AAGCTTCTATAGTTTGGCGTTCTTC	
Lcad-28a-F	GGATCCATGAGCATTGCACACGGA	OM909013; to establish the LCAD protein expression vector
Lcad-28a-R	AAGCTTCTACTGGCTGGGGTAGTC	

^*a*^ Underlining indicates restriction site of restriction endonuclease.

**TABLE 3:** Abbreviations appearing in this paper and their full terms

Abbreviations	Full terms	Abbreviations	Full terms
ABA	Abscisic acid	LCAD	Long chain acyl-CoA dehydrogenase
Ac-CoA	Acetyl coenzyme A	MDH	Malate dehydrogenase
ADC	Arginine decarboxylase	mICDH	Mitochondrial isocitrate dehydrogenase
ATP	Adenosine triphosphate	Mpc	Mitochondrial pyruvate carrier
α-KGDH	α-ketoglutarate dehydrogenase	M/SCAD	Medium/short-chain acyl-CoA dehydrogenase
CA	Citric acid	NAD	Nicotinamide adenine dinucleotide
CACT	Carnitine acylcarnitine translocase	NADH	Reduced nicotinamide adenine dinucleotide
CPT	Carnitine palmitoyl-transferase	OCR	Oxygen consumption rate
CS	Citrate synthase	ODC	Ornithine decarboxylase
ECH	Enoyl-CoA hydratase	OXPHOS	Oxidative phosphorylation
ETC	Electron transport chain	PFK	Phosphofructokinase
FAD	Flavin adenine dinucleotide	PK	Pyruvate kinase
FADH_2_	Reduced flavin adenine dinucleotide	ROS	Reactive oxygen species
GABA	γ-aminobutyric acid	SAMS	S-adenosylmethionine synthetase
GAPDH	Glyceraldehyde 3-phosphate dehydrogenase	SAMDC	S-adenosylmethionine decarboxylase
GAs	Ganoderic acids	SDH	Succinate dehydrogenase
HADH	Mitochondrial trifunctional protein (also known as MTP)	Spd	Spermidine
HADHA	Mitochondrial trifunctional protein α subunit	SPDS	Spermidine synthase
HCD	β-hydroxyacyl CoA dehydrogenase	TCA	Tricarboxylic acid
KCT	3-ketoacyl-CoA thiolase	WT	Wild-type

### Statistical analysis

To ensure the reproducibility of trends and relationships, three independent biological samples were prepared, with each sample tested in triplicate. The average of three replicate tests was taken as a single experimental data point. The standard deviation, represented by error bars, was calculated from the three independent experimental data points. Differences in mean values between groups were analyzed by one-way analysis of variance followed by Duncan’s multiple-range test. Different letters indicate significant differences between groups (*P* < 0.05).

## Data Availability

The data that support the findings of this study are available from the corresponding author upon reasonable request. The nucleotide sequences used in this study from *G. lucidum* strain ACCC53264 have been deposited in GenBank under accession numbers OM909012 (*hadhA*) and OM909013 (*lcad*).

## SUPPLEMENTAL MATERIAL

Supplemental material aem.00979-24-s0001.docx Figures S1 to S7, Table S1, and supplemental methods.

ASM does not own the copyrights to Supplemental Material that may be linked to, or accessed through, an article. The authors have granted ASM a non-exclusive, world-wide license to publish the Supplemental Material files. Please contact the corresponding author directly for reuse.
