# Supplementary material for: Spermidine enhances the heat tolerance of Ganoderma lucidum by promoting mitochondrial respiration driven by fatty acid β-oxidation
Source: Appl Environ Microbiol. 2025 Jan 29;91(2):e00979-24. doi: 10.1128/aem.00979-24 (PMC11837530; doi:10.1128/aem.00979-24)
Supplement: Supplemental material — Figures S1 to S7, Table S1, and supplemental methods. [file aem.00979-24-s0001.docx]

**Supplemental Materials**

**Spermidine enhances the heat tolerance of *Ganoderma lucidum* by promoting mitochondrial respiration driven by fatty acid β-oxidation**

Xiaofei Han,^1, 2^ Zi Wang,^1^ Lingyan Shi,^1^ Ziyang Wei,^1^ Jiaolei Shangguan,^1^ Liang Shi,* ^1^ Mingwen Zhao* ^1^

^1^ Key Laboratory of Agricultural Environmental Microbiology, Ministry of Agriculture, Department of Microbiology, College of Life Sciences, Nanjing Agricultural University, Nanjing, Jiangsu, 210095, China.

^2^ School of Medicine, Henan Polytechnic University, Jiaozuo, Henan, 454000, China.

*For correspondence.

E-mail: [shiliang@njau.edu.cn,](mailto:shiliang@njau.edu.cn;) mwzhao@njau.edu.cn; Tel./Fax 0086-25-84395602

**Supplemental Results**

**Fig. S1**


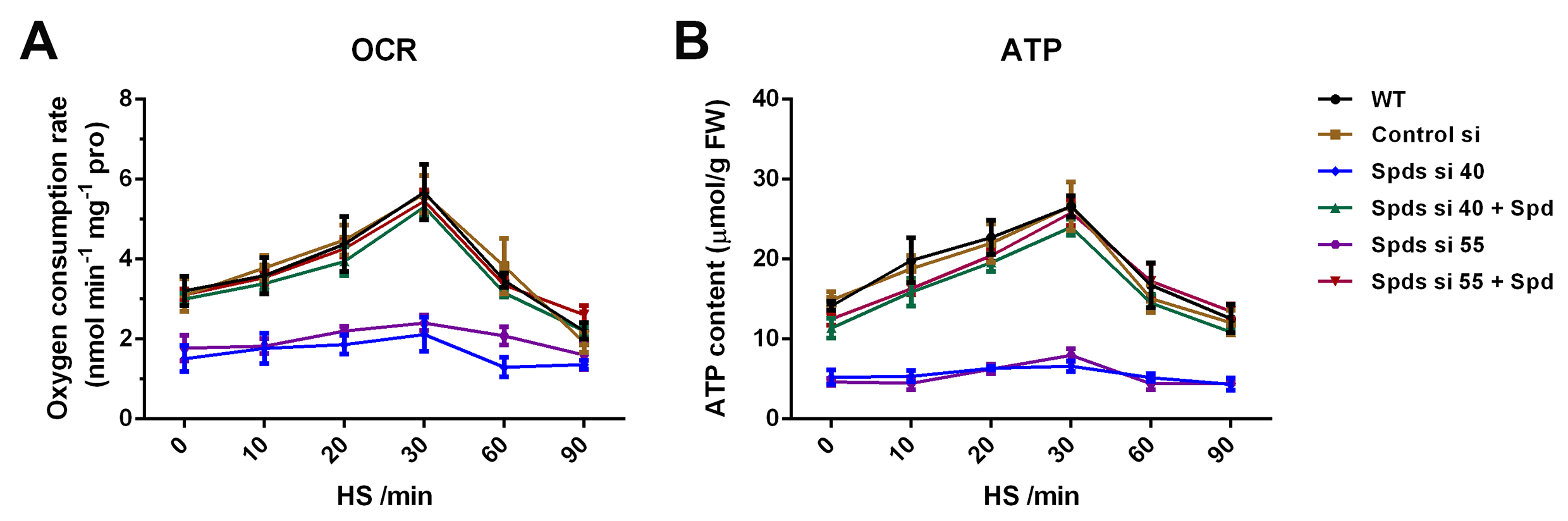


**Fig. S1.** Analysis of the respiratory level of *G. lucidum* strains under heat stress. (A) The oxygen consumption rate (OCR) in WT, Control si, *spdS* knockdown strains, and strains supplemented with 1 mM Spd under different heat stress times grown in CYM liquid medium. (B) The ATP levels in these strains under different heat stress times in CYM solid medium. The values presented are the mean ± standard deviation (SD) from three independent experiments.

**Fig. S2**


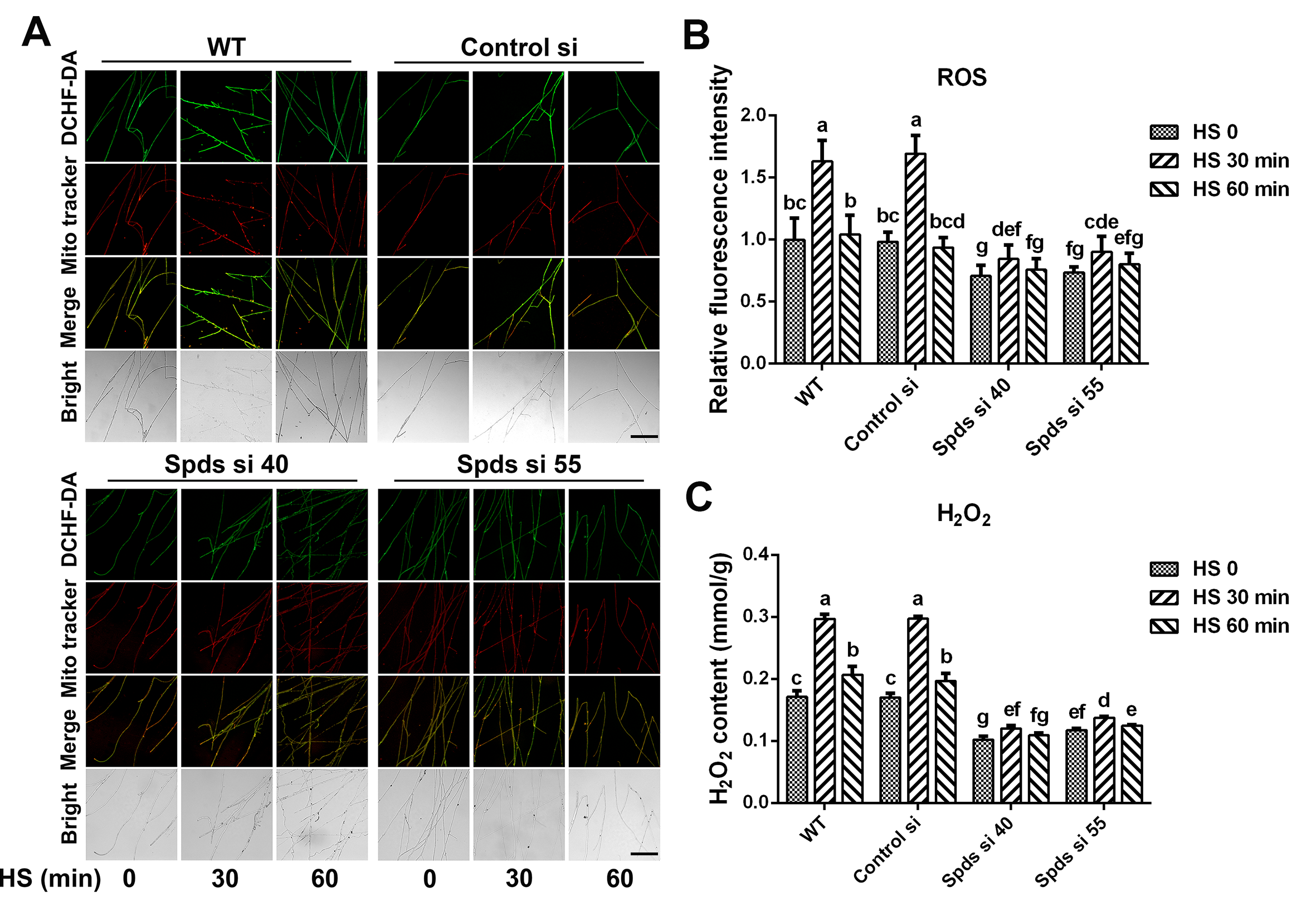


**Fig. S2.** Analysis of the ROS level of *G. lucidum* strains under heat stress. (A) The mitochondrial ROS levels determined by DCHF-DA and MitoTracker Red double staining in the WT, Control si, and *spdS* knockdown strains under heat stress in CYM solid medium. (B) Relative fluorescence intensities from panel A. (C) The H_2_O_2_ contents in the WT, Control si, and *spdS* knockdown strains under heat stress in CYM solid medium. The values presented are the mean ± standard deviation (SD) from three independent experiments. Different letters indicate significant differences between the lines (P<0.05, according to Duncan’s multiple-range test).

**Fig. S3**


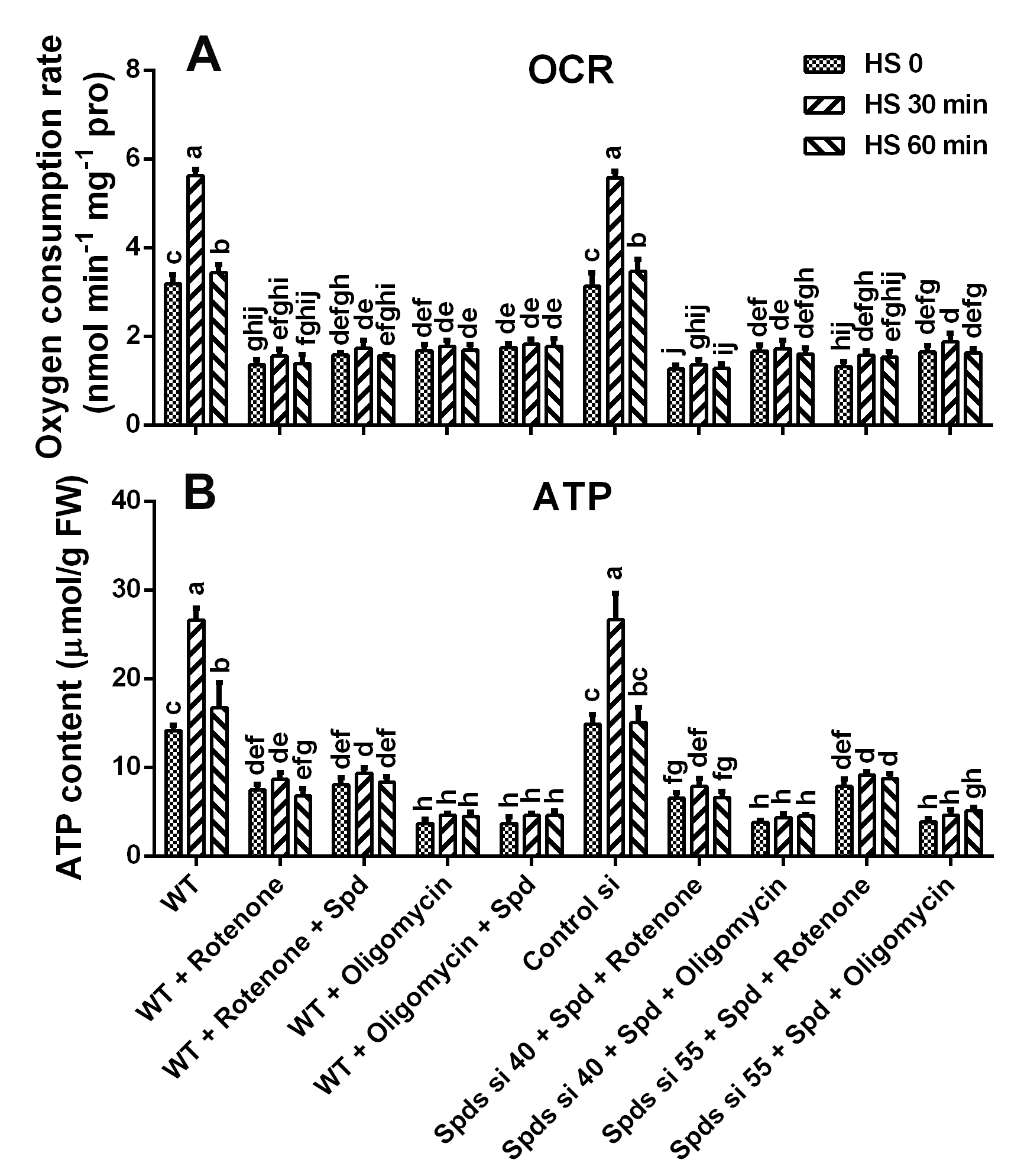


**Fig. S3.** The oxygen consumption rate and ATP contents of *G. lucidum* strains under heat stress after treatment with rotenone or oligomycin. (A) The oxygen consumption rate in WT, Control si, *spdS* knockdown strains, and strains treated with rotenone or oligomycin grown in CYM solid medium under heat stress. (B) The ATP contents in WT, Control si, *spdS* knockdown strains, and strains treated with rotenone or oligomycin in CYM solid medium under heat stress. The values presented are the mean ± standard deviation (SD) from three independent experiments. Different letters indicate significant differences between the lines (P<0.05, according to Duncan’s multiple-range test).

**Fig. S4**


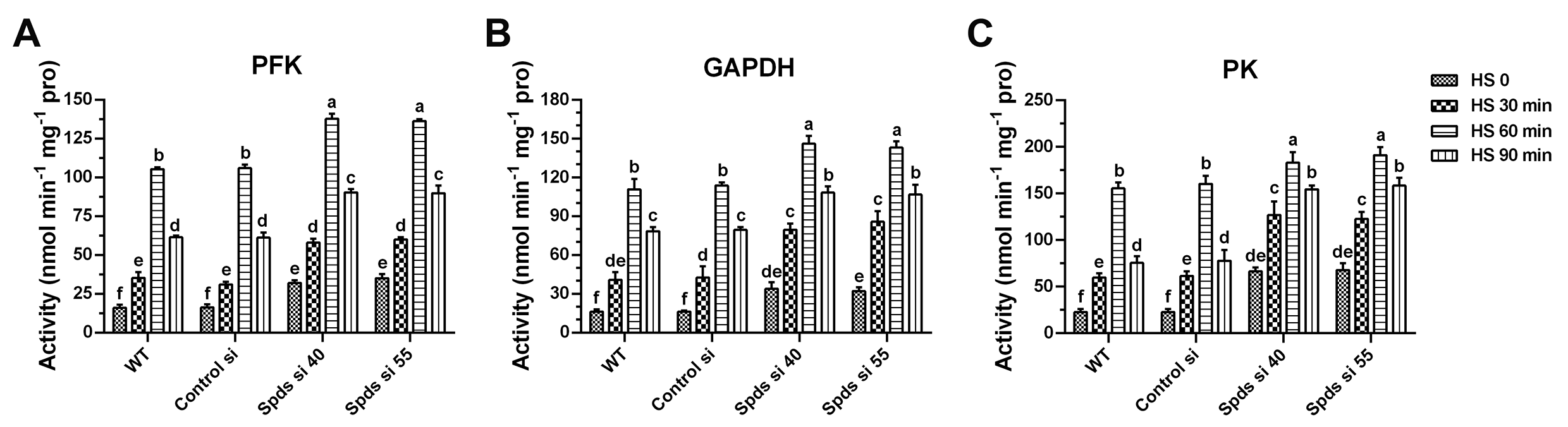


**Fig. S4.** Analysis of key enzymes activities in glycolysis of *G. lucidum* strains under heat stress. (A-C) Activities of phosphofructokinase (PFK), glyceraldehyde 3-phosphate dehydrogenase (GAPDH) and pyruvate kinase (PK) in WT, Control si, and *spdS* knockdown strains under heat stress in CYM solid medium. The values presented are the mean ± standard deviation (SD) from three independent experiments. Different letters indicate significant differences between the lines (P<0.05, according to Duncan’s multiple-range test).

**Fig. S5.**


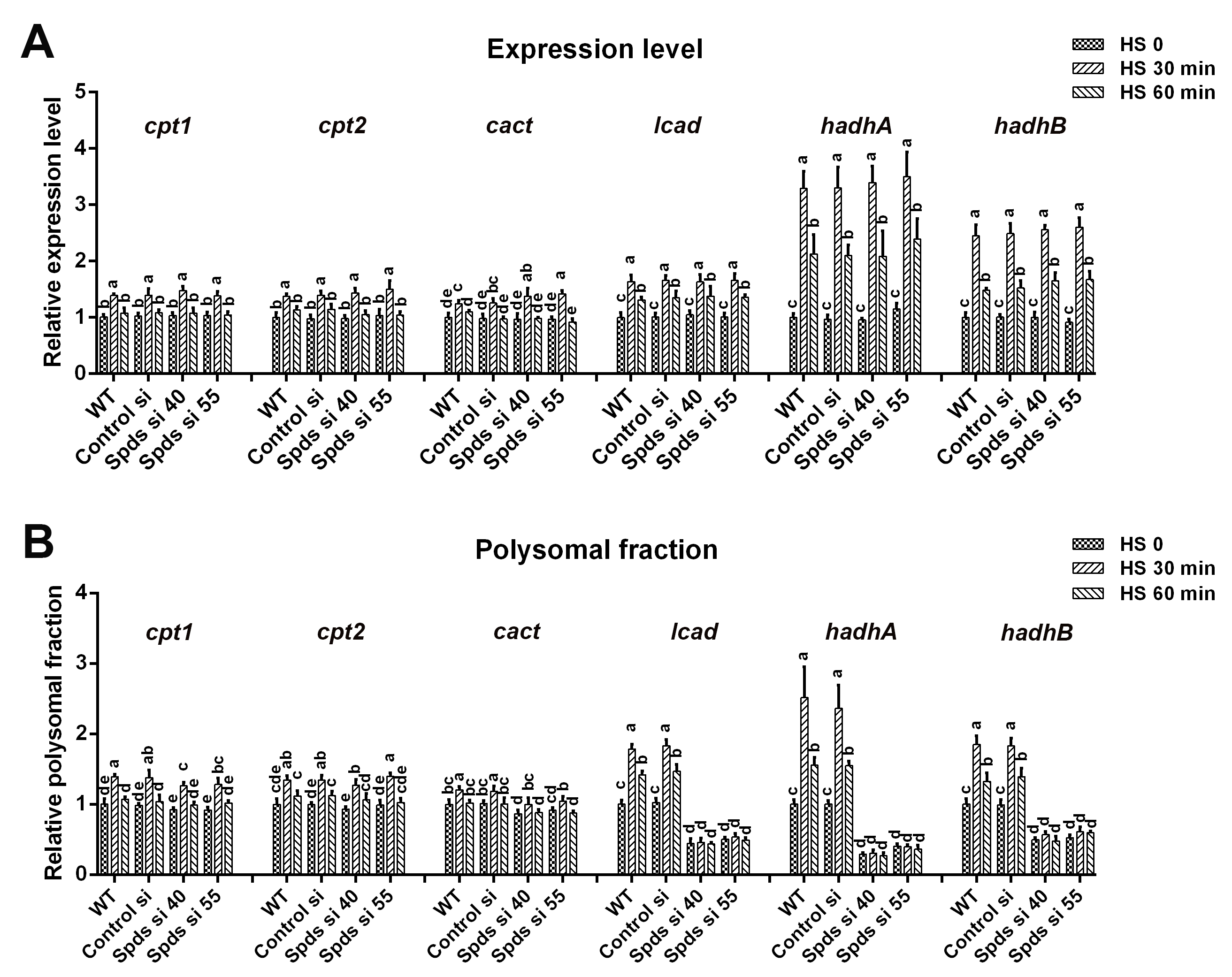


**Fig. S5.** Analysis of the gene expression levels and polysomal mRNA fractions related to long-chain fatty acid β-oxidation under heat stress. (A) The expression levels of genes related to long-chain fatty acid β-oxidation in WT, Control si, and *spdS* knockdown strains under heat stress in CYM solid medium. (B) The polysomal mRNA fractions related to long-chain fatty acid β-oxidation in WT, Control si, and *spdS* knockdown strains under heat stress in CYM solid medium. The values presented are the mean ± standard deviation (SD) from three independent experiments. Different letters indicate significant differences between the lines (P<0.05, according to Duncan’s multiple-range test).

**Fig. S6**


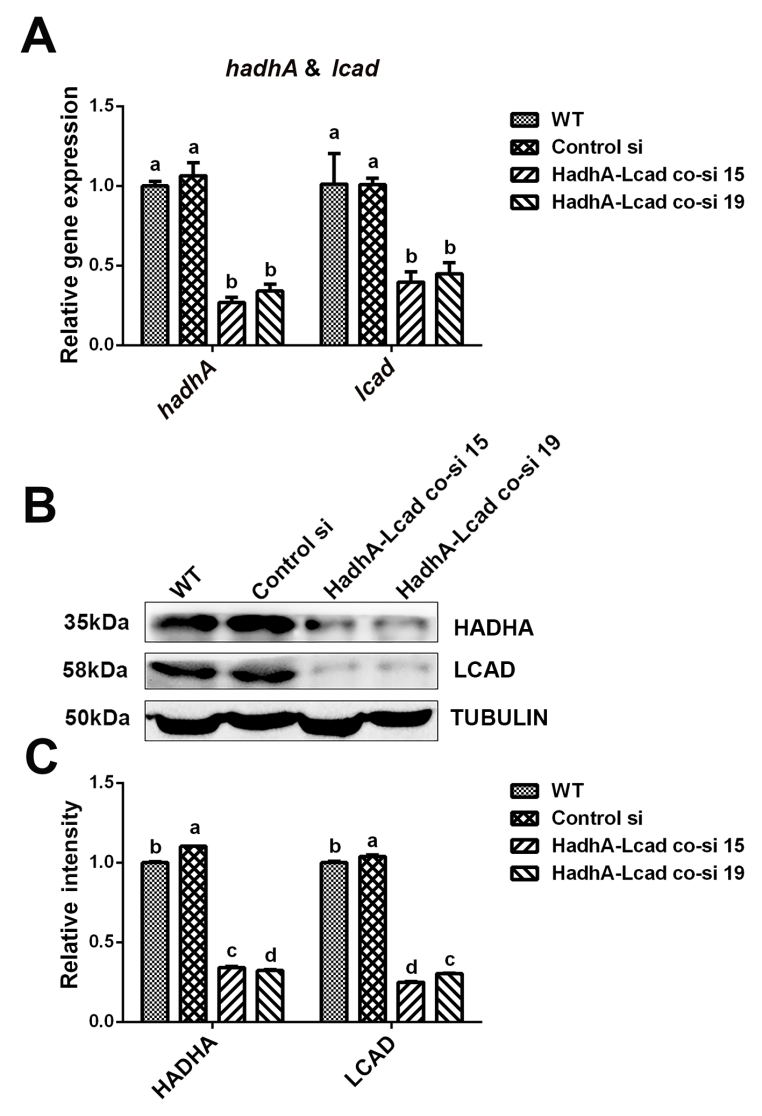


**Fig. S6.** Establishment of *lcad* and *hadhA* co-knockdown strains. (A) The expression levels of *lcad* and *hadhA* in WT, Control si, *lacd* and *hadhA* co-knockdown strains in CYM solid medium. (B) LCAH and HADH protein levels in WT, Control si, *lacd* and *hadhA* co-knockdown strains in CYM solid medium detected by Western blot. (C) The relative intensity from Panel B. The values presented are the mean ± standard deviation (SD) from three independent experiments. Different letters indicate significant differences between the lines (P<0.05, according to Duncan’s multiple-range test).

**Fig.S7**


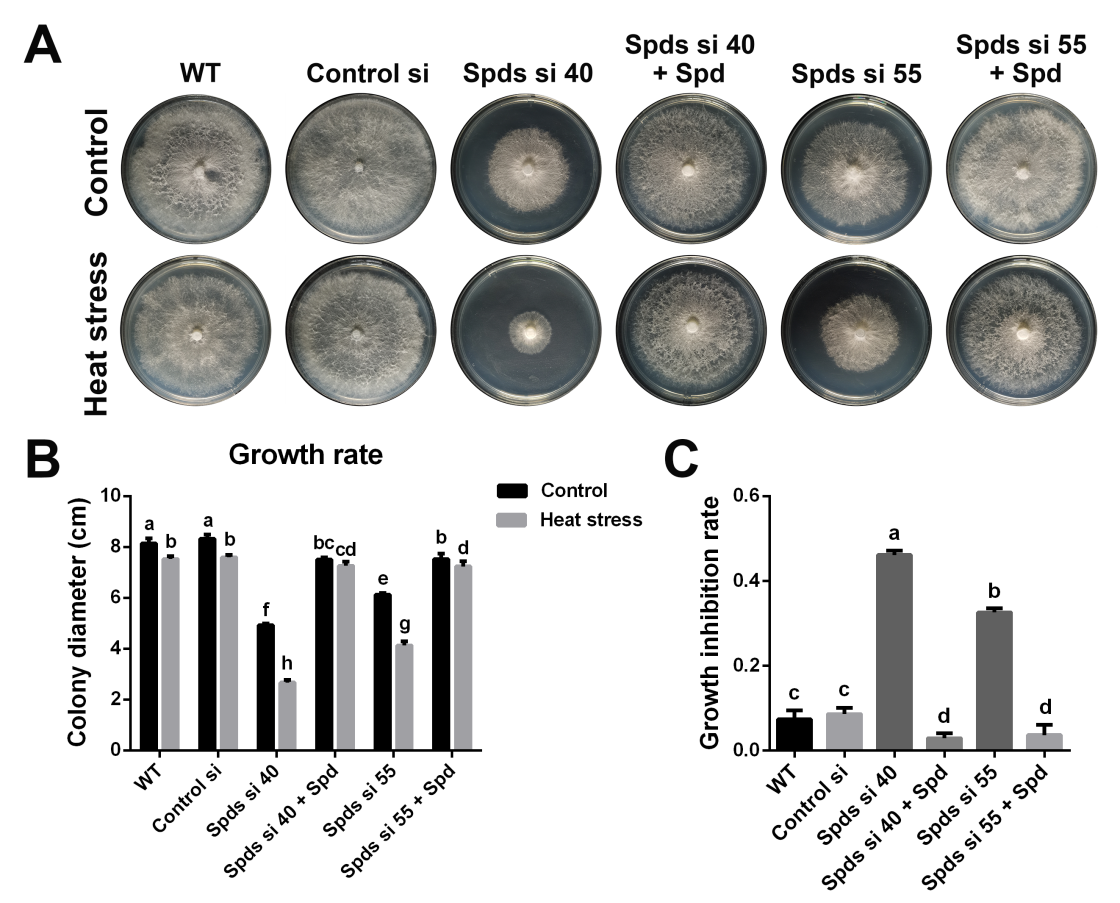


**Fig. S7.** Analysis of the heat tolerance of *G. lucidum* strains. (A) The growth status of WT, Control si, *spdS* knockdown strains, and strains supplemented with 1 mM Spd under heat stress in a polyamine-free medium (2% Glucose, 0.5% KHPO_4_, 0.1% MgSO_4_•7H_2_O, 0.8% Asparagine). (B) The colony diameter from Panel A. (C) The growth inhibition rate of these strains under heat stress. The values presented are the mean ± standard deviation (SD) from three independent experiments. Different letters indicate significant differences between the lines (P<0.05, according to Duncan’s multiple-range test).

**Table S1** The list of strains used in this study.

| **Strains** | **Descriptions** | **References** | **Accessions and Orthologs** |
| --- | --- | --- | --- |
| WT | Wild-type | *G. lucidum* strain ACCC53264 from the Agricultural Culture Collection of China | / |
| Control si | Transformed with an empty vector of gene knockdown vector | Tao *et al.* (2021) ^[1]^ | / |
| Spds si 40 | Spermidine synthase gene *spdS1* and *spdS2* co-knockdown | Tao *et al.* (2021), originally named GlSpds-kd40 ^[1]^ | Spermidine synthase (SpdS) [Accession: ULF48217 (SpdS1); ULF48218 (SpdS2)], ortholog from *Saccharomyces cerevisiae* (Accession: GFP66190) |
| Spds si 55 | Spermidine synthase gene *spdS1* and *spdS2* co-knockdown | Tao *et al.* (2021), originally named GlSpds-kd55 ^[1]^ |  |
| HadhA-Lcad co-si 15 | Long-chain acyl-CoA dehydrogenase gene (*lcad*) and mitochondrial trifunctional protein gene ( *hadhA* ) co-knockdown | This study | Long-chain acyl-CoA dehydrogenase (LCAD) (Accession: UOO00980), ortholog from homo sapiens (Accession: AAA51565) ^(a)^; Mitochondrial trifunctional protein (HADH) (Accession: UOO00979), ortholog from homo sapiens (Accession: AAH09235) ^(a)^. |
| HadhA-Lcad co-si 19 | Long-chain acyl-CoA dehydrogenase gene (*lcad*) and mitochondrial trifunctional protein gene ( *hadhA* ) co-knockdown | This study |  |

Note: (a) No ortholog found in typical model fungi, so an ortholog from homo sapiens was provided.

**Supplemental Method**

**Extraction and detection of polyamines**

Polyamine was extracted and detected as previously described ^[1]^. Briefly, 200 mg of fresh mycelia were homogenized in 2 mL of 5% (w/v) cold perchloric acid, incubated for 1 h on the ice, and then centrifuged to obtain the supernatant. 1 mL of 2 M NaOH was added to the supernatant to neutralize the perchloric acid, and then 10 μL of benzoyl chloride were subsequently added to the mixture and incubated for 30 min at 37°C to obtain benzoyl polyamines. 2 mL of saturated NaCl and 2 mL of diethyl ether were added to the mixture and centrifuged to obtain the supernatant diethyl ether phase. The 1 mL of the diethyl ether phase was evaporated to obtain the crude extract of benzoyl polyamines. The crude extract of benzoyl polyamines was dissolved in 0.5 mL of methanol and analyzed with HPLC (Agilent Technologies, Santa Clara, CA, USA).

**Detection of ROS and H_2_O_2_**

The level of ROS was assessed by fluorescence analysis according to a previous method ^[2]^. The mycelia were stained with 2′,7′-dichlorodihydro-fluorescein diacetate (DCHF-DA) for 20 min, Fluorescence was detected with a fluorescence microscope (Zeiss Axio Imager A1), and the fluorescence intensity was analyzed with ZEN lite software (Zeiss). Similarly, the mycelia were double-stained with DCFH-DA and Mito-Tracker Red to detect mitochondrial ROS, as described by Han ^[2]^. The mitochondria were stained with Mito-Tracker Red, and ROS was stained with DCHF-DA for 20 min. The fluorescence was detected with a fluorescence microscope, and the fluorescence intensity was analyzed with ZEN lite software. The content of intracellular H_2_O_2_ was detected with a hydrogen peroxide assay kit (Beyotime, China) according to the manufacturer’s protocol.

**Reference**

1. Tao YX, Han XF, Ren A, Li J, Song HB, Xie BG, Zhao MW. 2021. Heat stress promotes the conversion of putrescine to spermidine and plays an important role in regulating ganoderic acid biosynthesis in *Ganoderma lucidum*. Appl Microbiol Biotechnol 105:5039-5051.
2. Han XF, Shangguan JL, Wang Z, Li Y, Fan JP, Ren A, Zhao MW. 2022. Spermidine regulates mitochondrial function by enhancing eIF5A hypusination and contributes to reactive oxygen species production and ganoderic acids biosynthesis in *Ganoderma lucidum*. Appl Environ Microbiol 88: e02037-21.
